# Seladelpar Improved Itch, Itch‐Related Sleep Disturbance and Measures of Fatigue in Patients With Primary Biliary Cholangitis and Pruritus in the Phase 3 RESPONSE Trial

**DOI:** 10.1111/apt.70630

**Published:** 2026-04-03

**Authors:** Cynthia Levy, David E. J. Jones, Marlyn J. Mayo, Christopher L. Bowlus, Kris V. Kowdley, Gideon M. Hirschfield, Jie Liu, Caroline Burk, Susheela Carroll, Daria B. Crittenden, Andreas E. Kremer

**Affiliations:** ^1^ Schiff Center for Liver Diseases University of Miami Miller School of Medicine Miami Florida USA; ^2^ Division of Digestive Health and Liver Diseases University of Miami School of Medicine Miami Florida USA; ^3^ Department of Medical Sciences NIHR Newcastle Biomedical Research Centre Newcastle UK; ^4^ Division of Digestive and Liver Diseases University of Texas SW Medical Center Dallas Texas USA; ^5^ Division of Gastroenterology and Hepatology University of California Davis School of Medicine Sacramento California USA; ^6^ Liver Institute Northwest Seattle WA USA; ^7^ The Autoimmune and Rare Liver Disease Programme, Toronto General Hospital Toronto Ontario Canada; ^8^ Gilead Sciences, Inc. Foster City California USA; ^9^ Department of Gastroenterology and Hepatology, University Hospital Zürich University of Zürich Zürich Switzerland

**Keywords:** cholestatic liver disease, liver, outcomes research, primary biliary cholangitis

## Abstract

**Background:**

Pruritus and fatigue impose a significant burden on patient quality of life in primary biliary cholangitis (PBC). In the Phase 3 RESPONSE trial (NCT04620733), seladelpar, a potent and selective PPARδ agonist, significantly improved markers of cholestasis and pruritus.

**Aims:**

Here, we provide additional details on the effects of seladelpar on pruritus and quality of life.

**Methods:**

In RESPONSE, patients with PBC and an inadequate response or intolerance to ursodeoxycholic acid were randomised 2:1 to seladelpar 10 mg or placebo for 12 months. Changes in severity of pruritus by the numeric rating scale (NRS) and changes in 5‐D Itch and PBC‐40 in patients with moderate to severe itch (NRS ≥ 4), severe itch (NRS ≥ 7) and clinically significant itch (PBC‐40 itch ≥ 7) at baseline were evaluated.

**Results:**

In patients with baseline NRS ≥ 4, mean NRS improved from the moderate to mild range with seladelpar, but not placebo. Bodily distribution of itch was reduced with seladelpar based on the 5‐D itch distribution domain. Itch‐related sleep disturbance was consistently improved with seladelpar across populations, and measures of fatigue were improved versus placebo in patients with severe itch at baseline. Patients without itching tended to remain free from itching with seladelpar, consistent with fewer adverse events of pruritus with seladelpar versus placebo.

**Conclusion:**

In addition to an established anticholestatic effect, seladelpar improved pruritus severity and distribution, itch‐related sleep disturbance and measures of fatigue in patients with PBC and pruritus, indicating clinically relevant improvements in itch and other quality of life benefits.

## Graphical abstract

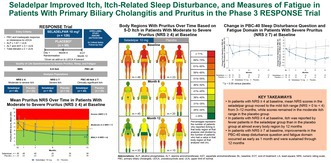

## 1 Introduction

Primary biliary cholangitis (PBC) is a chronic, progressive, autoimmune, cholestatic liver disease that affects approximately 1 in 1000 women over 40 years of age [1]. PBC is characterised by immune‐mediated bile duct destruction, leading to cholestasis and accumulation of toxic bile acids [1, 2, 3, 4]. This can result in liver injury and fibrosis, progression to cirrhosis, end‐stage liver disease and death [1, 2, 3].

Most people with PBC experience pruritus and fatigue during their course of disease [3, 5], though both symptoms are likely underrecognised in the clinic [6]. Pruritus can profoundly compromise quality of life, as many patients report substantial disability associated with their pruritus, affecting social interactions, household responsibilities and occupational or academic performance [7, 8]. In the most severe cases of therapy‐refractory itching, cholestatic pruritus may become an indication for liver transplantation, even in the absence of liver failure [6]. Fatigue is a very common comorbidity in PBC that is likely multifactorial in nature, although pruritus can worsen fatigue due to its impact on sleep [3, 5].

Seladelpar is a first‐in‐class delpar (potent and selective peroxisome proliferator–activated receptor delta [PPARδ] agonist) indicated for the treatment of PBC in combination with ursodeoxycholic acid (UDCA) in adults who have an inadequate response to UDCA or as monotherapy in patients who are unable to tolerate UDCA [9, 10, 11]. In the randomised, placebo‐controlled, Phase 3 RESPONSE trial (NCT04620733), seladelpar treatment resulted in significant improvements in cholestatic markers of disease as well as a clinically and statistically significant reduction of pruritus at 6 months in patients who had moderate to severe pruritus (numeric rating scale [NRS] score of ≥ 4) at baseline, meeting the key secondary endpoint of the study [12]. This effect was sustained through 12 months of treatment [12]. Results of the 5‐D Itch scale and PBC‐40 itch domain also demonstrated improvements with seladelpar treatment in the NRS ≥ 4 population [12].

Here, we present detailed post hoc analyses from the pivotal, Phase 3 RESPONSE trial, assessing changes in pruritus and its impact on sleep, as well as impacts of fatigue and other health‐related quality of life measures in greater depth. These analyses aimed to provide a more comprehensive understanding of the clinical effects of seladelpar in patients with PBC and pruritus and to further evaluate symptom burden and quality of life changes that are meaningful to patients.

## 2 Methods

### 2.1 Study Design and Participants

RESPONSE was a Phase 3, multicentre, double‐blind, randomised, placebo‐controlled trial in patients with PBC who had an inadequate response to UDCA after at least 12 months of treatment or who were intolerant to UDCA [12]. Patients were randomly assigned (2:1) to oral seladelpar 10 mg or placebo daily for 12 months along with UDCA if tolerant (Figure S1) [12]. Antipruritic medications were allowed if patients were on a stable dose for at least 1 month prior to screening [12]. A report of the RESPONSE trial following the CONSORT reporting guidelines, including full methodology and eligibility criteria, was previously published [12]. Written consent was obtained from all patients before screening. This study was approved by regulatory authorities and institutional review board or ethics committees at each site and was conducted in strict accordance with the Declaration of Helsinki and International Conference on Harmonisation Good Clinical practise guidelines.

During RESPONSE, patient‐reported outcomes (PROs) of pruritus and other quality of life measures were collected via the pruritus NRS, the 5‐D Itch scale and the PBC‐40. To further characterise effects of seladelpar on pruritus in the RESPONSE trial, patient populations were analysed based on their baseline itch status, including patients who had moderate to severe (NRS ≥ 4) or severe (NRS ≥ 7) pruritus based on the pruritus NRS [13]. Patients with clinically significant pruritus at baseline based on the PBC‐40 (PBC‐40 itch ≥ 7) were also assessed [7], which allowed for another opportunity to assess the consistency of the effect of seladelpar using a PBC‐specific quality of life scale. Lastly, patients with no or near no itch (NRS = 0–1) at baseline in the RESPONSE trial were evaluated.

### 2.2 Pruritus and Quality of Life Scales

Pruritus NRS data were collected daily from the run‐in visit through 6 months and then for 7 consecutive days during each month through the end of the 12‐month treatment period (Figure S1) [12]. The Worst Itch NRS is a well‐defined, reliable, and sensitive scale for evaluating the intensity of pruritus that has been validated for use in PBC [14, 15]. Patients rate their worst itch in the past 24 h on a scale of 0 (no itch) to 10 (worst itch imaginable) [12]. An NRS score of ≥ 4 represents moderate to severe pruritus [13]. A ≥ 3‐point improvement in the NRS score represents a clinically meaningful change in patients with moderate to severe pruritus at baseline [16].

5‐D Itch data were collected every 2 weeks from run‐in through 6 months, then monthly through 12 months or until the end of treatment (Figure S1) [12]. The 5‐D Itch scale measures the impact of itching on people's lives (Table S1) [17]. This tool assesses the duration (hours per day), degree (severity), direction (improvement or worsening), disability (four items addressing impact of itch on sleep [falling asleep/night awakenings], leisure/social, housework/errands and work/school) and distribution (16 potential affected body regions; each scored with ‘absent’ or ‘present’) of itch over the past 2 weeks. All domains are scored on a scale of 1 to 5, with higher scores indicating greater impairment [17, 18].

PBC‐40 data were collected at run‐in, randomisation, 1 month, 3 months and then every 3 months through 12 months or until the end of treatment (Figure S1) [12]. The PBC‐40 is a patient‐derived, disease‐specific quality of life questionnaire used in clinical PBC studies that assesses six domains: itch (including a question for how itching disturbed sleep), fatigue, cognitive, and symptoms over the last 4 weeks, along with social and emotional overall/in general (Table S2) [19]. Scores on the PBC‐40 itch domain, sleep disturbance question (within the itch domain) and fatigue domain ranged from 0 to 15, 0 to 5 and 11 to 55, respectively, with higher scores indicating greater impairment [19, 20]. A PBC‐40 itch domain score of ≥ 7 at baseline, termed here as patients with clinically significant pruritus [7], and a PBC‐40 fatigue domain score of ≥ 29 represents moderate to severe fatigue [20]. A clinically meaningful change in the PBC‐40 domain score was defined as a 0.5‐point change from baseline per item [21].

### 2.3 Patient Populations and Outcomes Assessed

Table S3 summarises the health‐related quality of life outcomes reported here by analysis population and notes pruritus and related outcomes that have been previously published. Details on the current analyses are as follows:

#### 2.3.1 Patients With Moderate to Severe Pruritus at Baseline (NRS ≥ 4)

Mean NRS values over time for patients with NRS ≥ 4 at baseline were evaluated to further characterise severity of pruritus. Improvements in the NRS by ≥ 3 and ≥ 4 points at 6 and 12 months in patients with NRS ≥ 4 were assessed. Near resolution of itch by NRS (NRS = 0–1) was also analysed over time. 5‐D Itch mean changes over time for all domains and the sleep item are summarised here at 6 and 12 months. Additional information on the duration of itch and regions of the body impacted by pruritus based on the 5‐D Itch duration and distribution domains is also reported. Change in all PBC‐40 domains including sleep disturbance at 6 and 12 months is reported. A Spearman rank correlation was conducted to investigate the relationship between the change in PBC‐40 itch and PBC‐40 fatigue domain scores at 6 and 12 months among patients with NRS ≥ 4 at baseline [22].

#### 2.3.2 Patients With Severe Pruritus at Baseline (NRS ≥ 7)

To explore the effects of seladelpar in patients with more severe pruritus at baseline, mean changes from baseline in the NRS among the NRS ≥ 7 population were assessed. Near resolution of itch by NRS (NRS = 0–1) was also assessed over time. 5‐D Itch and PBC‐40 data for all domains and the sleep item/question are summarised at 6 and 12 months.

#### 2.3.3 Patients With Clinically Significant Pruritus at Baseline (As Defined by PBC‐40 Itch ≥ 7)

Changes in PBC‐40 for all domains and the sleep disturbance question were assessed in patients with clinically significant itch at baseline at 6 and 12 months, as was the proportion of patients shifting from PBC‐40 itch ≥ 7 to < 7 with seladelpar versus placebo at 12 months.

#### 2.3.4 Overall Population and Patients With No or Near No Itch at Baseline (NRS = 0–1)

To evaluate patterns of change in NRS across the entire study population, a Sankey plot was generated to show shifts from baseline to 12 months in pruritus NRS by category of no or near no itch (NRS = 0–1), mild (NRS > 1 to < 4), moderate (NRS 4 to < 7), or severe itch (NRS ≥ 7). Patients with no or near no itch (NRS = 0–1) were also specifically evaluated for the development of itch, defined as an NRS increase to ≥ 2 points at 12 months.

### 2.4 Safety

Safety was assessed by treatment‐emergent adverse events (AEs; events occurring after initiation of study drug and within 30 days of last dose) in patients with moderate to severe itching (NRS ≥ 4) versus those with mild or no itching (NRS < 4) at baseline.

### 2.5 Statistical Analyses

Baseline pruritus NRS scores were defined as the mean of all daily recorded scores during the run‐in period and on day 1.5‐D Itch and PBC‐40 baseline scores were defined as the arithmetic mean of applicable measurements at screening, run‐in, day 1, and unscheduled assessments prior to or on day 1.

Responder analyses were conducted when assessing the percentage of patients achieving a binary outcome (e.g., NRS ≥ 3‐point decline, NRS ≥ 4‐point decline) as described above. In all responder analyses, patients with missing data at specific time points were considered nonresponders. Least‐square (LS) mean differences over time in the 5‐D Itch domains/sleep item and PBC‐40 domains/sleep disturbance question were estimated by mixed‐effects models for repeated measures. For the Sankey plot of the overall population, patients with both baseline and 12‐month NRS values were included.

All analyses not previously reported were post hoc in nature; thus, displays of statistical significance should be interpreted as descriptive in nature. Reported AEs were summarised by counts and percentages. All analyses were conducted using SAS version 9.4 (Cary, NC, USA).

## 3 Results

### 3.1 Patients With Moderate to Severe Pruritus (NRS ≥ 4) at Baseline

In the RESPONSE trial, 128 patients received seladelpar and 65 patients received placebo [12]. The details of patient disposition of this study, including the 90.2% (*n* = 174) of patients who completed the study, were previously reported [12]. Of the enrolled patients, 49 in the seladelpar group and 23 in the placebo group had NRS ≥ 4 at baseline (Table 1). The mean age at diagnosis of those with NRS ≥ 4 was 47 years, and the majority of patients were female and White. Most patients with NRS ≥ 4 at baseline had a reported history of fatigue (63%, seladelpar; 70%, placebo). Mean NRS score at baseline in the NRS ≥ 4 population was 6.1 in the seladelpar group and 6.6 in the placebo group, and mean PBC‐40 itch domain score was 8.7 in the seladelpar group and 9.6 in the placebo group. Of the patients with NRS ≥ 4 at baseline, 31 in the seladelpar group and 17 in the placebo group also had moderate to severe fatigue, defined as PBC‐40 fatigue domain score ≥ 29 at baseline (Table 1). Mean PBC‐40 fatigue domain scores were 31.9 and 34.7 in the NRS ≥ 4 seladelpar and placebo groups, respectively. At baseline, 22% of patients in both these groups were receiving concomitant antipruritic medications, which included cholestyramine, colestipol, rifampicin, gabapentin and sertraline. Among patients with NRS ≥ 4 at baseline, the rates of starting or stopping antipruritic medications during the 12‐month study were balanced between groups and generally infrequent. In the seladelpar group, two patients (4%) started and three patients (6%) stopped an antipruritic medication, compared with one patient (4%) who started and one (4%) who stopped in the placebo arm. Overall, 59 (82%) patients with NRS ≥ 4 at baseline completed the study, including 41 (84%) patients in the seladelpar arm and 18 (78%) in the placebo arm.

**TABLE 1:** Demographics and baseline clinical characteristics in patients with NRS ≥ 4 at baseline.

Characteristic	Seladelpar (*n* = 49)	Placebo (*n* = 23)
Age, years, mean (SD)	53 (10.7)	55 (10.3)
Age at PBC diagnosis, years, mean (SD)	47 (10.8)	47 (10.2)
Diagnosed at < 50 years, *n* (%)	24 (49)	12 (52)
Diagnosed at ≥ 50 years, *n* (%)	25 (51)	11 (48)
Female, *n* (%)	48 (98)	22 (96)
Race, *n* (%)		
American Indian or Alaska Native	2 (4)	2 (9)
Asian	1 (2)	1 (4)
Black or African American	1 (2)	0
White	44 (90)	20 (87)
Missing	1 (2)	0
Ethnicity, *n* (%)		
Hispanic or Latino	16 (33)	9 (39)
Not Hispanic or Latino	32 (65)	14 (61)
Missing	1 (2)	0
Duration of PBC, years, mean (SD)	7 (4.7)	9 (7.8)
History of fatigue, *n* (%)	31 (63)	16 (70)
Prior use of OCA/fibrates, *n* (%)	11 (22)	6 (26)
Any pruritus medication at baseline^a^, *n* (%)	11 (22)	5 (22)
Cholestyramine	5 (10)	3 (13)
Colestipol	0	1 (4)
Rifampicin	3 (6)	1 (4)
Gabapentin	2 (4)	0
Sertraline	3 (6)	0
UDCA intolerant, *n* (%)	4 (8)	1 (4)
NRS score, mean (SD)	6.1 (1.4)	6.6 (1.4)
PBC‐40		
Itch domain score, mean (SD)	8.7 (2.7)	9.6 (2.6)
Sleep question score, mean (SD)	3.2 (0.9)	3.4 (0.9)
Fatigue domain score, mean (SD)	31.9 (8.3)	34.7 (9.0)
Moderate to severe fatigue (PBC‐40 ≥ 29), *n* (%)	31 (63)	17 (74)
Cirrhosis, *n* (%)	6 (12)	5 (22)
ALP^b^, U/L, mean (SD)	338 (137)	341 (127)
ALT^c^, U/L, mean (SD)	51 (24)	54 (22)
Total bilirubin^d^, mg/dL, mean (SD)	0.82 (0.32)	0.71 (0.27)
GGT^e^, U/L, mean (SD)	287 (252)	317 (250)

Abbreviations: ALP, alkaline phosphatase; ALT, alanine aminotransferase; GGT, gamma‐glutamyl transferase; NRS, numeric rating scale; OCA, obeticholic acid; PBC, primary biliary cholangitis; UDCA, ursodeoxycholic acid; ULN, upper limit of normal.

^a^ Details regarding pruritus medications are expanded upon in the Supplemental Methods.

^b^ The ULN is 116 U/L in men and women.

^c^ The ULN is 41 U/L in men and women.

^d^ The ULN is 1.10 mg/dL in men and women.

^e^ The ULN is 52 U/L in men and 38 U/L in women.

**FIGURE 1:**
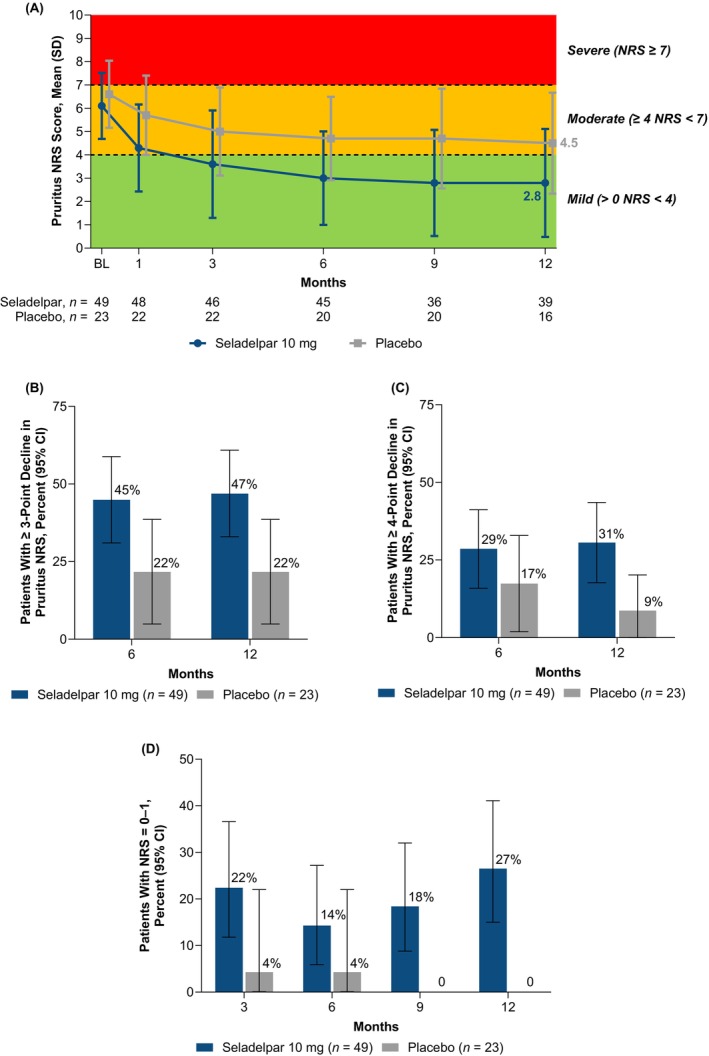
Mean pruritus NRS over time (A), percentage of patients with a ≥ 3‐point NRS decline at 6 and 12 months (B), percentage of patients with a ≥ 4‐point NRS decline at 6 and 12 months (C) and percentage of patients with near resolution of itch (NRS = 0–1) (D) in patients with moderate to severe pruritus based on NRS ≥ 4 at BL. A colour‐blind accessible version of panel (A) is available in Figure S8. In panel (A), *n* = the number of patients who had both a BL value and a value at that time point. In panels (B–D), patients with missing data at the specific time point were considered nonresponders. BL, baseline; NRS, numeric rating scale.

Consistent with achieving a statistically significant reduction from baseline in the pruritus NRS score at 6 months among patients with NRS ≥ 4 at baseline (key secondary endpoint in RESPONSE) [12], mean NRS for patients treated with seladelpar moved from the moderate itch range (NRS ≥ 4 to < 7) at baseline to the mild itch range (NRS > 0 to < 4) from 3 months through 12 months, while those on placebo remained in the moderate itch range throughout the duration of the study (Figure 1A). For patients with NRS ≥ 4 at baseline, 47% of patients on seladelpar demonstrated a ≥ 3‐point decrease in their pruritus NRS score versus 22% on placebo at 12 months (Figure 1B); a ≥ 4‐point decrease in NRS was achieved by 31% of patients on seladelpar compared with 9% of patients on placebo (Figure 1C). Among patients who entered the study with NRS ≥ 4, 27% who received seladelpar demonstrated near resolution of itch (NRS = 0–1) at 12 months compared with 0% in the placebo group (Figure 1D).

**FIGURE 2:**
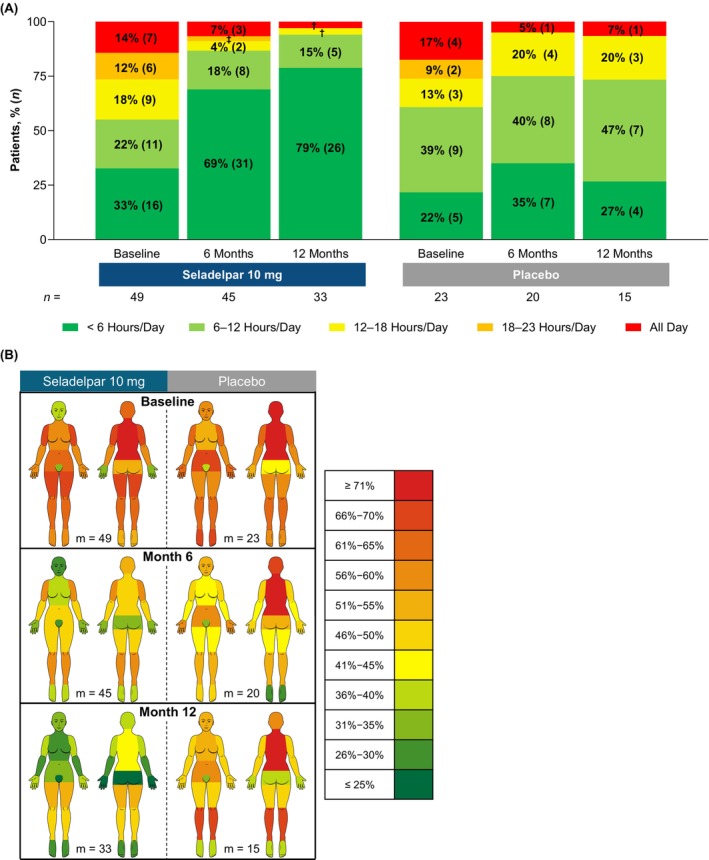
Daily duration of itch (A) and body regions with pruritus (B) over time based on 5‐D Itch in patients with moderate to severe pruritus based on NRS ≥ 4 at BL. Colour‐blind accessible versions of panels (A) and (B) are available in Figure S9. ^‡^In panel (A), the symbol represents 2% (*n* = 1). ^†^In panel (A), the symbol represents 3% (*n* = 1). In panel (A), *n* = the number of patients who had both a BL value and a value at that time point. In panel (B), percentages represent the number of patients who reported itching in that body region at that analysis visit divided by the number of patients who had a value in that body region at that analysis visit (m). BL, baseline; NRS, numeric rating scale.

In patients with NRS ≥ 4 at baseline, reductions in 5‐D Itch score over the 12‐month treatment period were greater overall among patients treated with seladelpar than placebo [12]; changes in all domains and the sleep disturbance question at 6 and 12 months are summarised in Figure S2. Per 5‐D Itch duration domain data, seladelpar reduced the number of hours spent itching per day at 6 months and 12 months when compared with placebo (Figure 2A). A body map analysis of the 5‐D Itch distribution domain (Figure 2B) was used to portray the percentage of patients who reported itching at various body regions over time. By 12 months, itch was reported by fewer patients in the seladelpar group than in the placebo group at almost every body region, including the head (13/33 [39%] vs. 9/15 [60%] for seladelpar vs. placebo) and back (15/33 [45%] vs. 12/15 [80%]), among others.

Numerical reductions from baseline in the PBC‐40 itch domain and sleep disturbance question were greater with seladelpar versus placebo [12]; changes in all domains and the sleep disturbance question at 6 and 12 months are summarised in Figure S3.

In addition to the itch domain and the itch‐related sleep disturbance question, most other domains in the PBC‐40, including fatigue, had mean score changes that favoured seladelpar. There was a moderate, positive correlation between change in PBC‐40 pruritus and PBC‐40 fatigue domain scores at 6 months (*n* = 59; *ρ* = 0.37; *p* = 0.0041) and 12 months (*n* = 52; *ρ* = 0.32; *p* = 0.0209; Figure S4).

In patients with NRS ≥ 4 and moderate to severe fatigue (PBC‐40 fatigue ≥ 29) at baseline, a similar trend was observed with numerical reduction in mean fatigue scores for seladelpar versus placebo (LS mean difference [CI] was −2.0 [−5.7, 1.8] at 6 months and −3.1 [−7.7, 1.6] at 12 months).

### 3.2 Patients With Severe Pruritus (NRS ≥ 7) at Baseline

Sixteen patients (13%) in the seladelpar group and 12 patients (18%) in the placebo group had NRS ≥ 7 at baseline. Further demographic and baseline clinical characteristics for this group are reported in Table S4.

**FIGURE 3:**
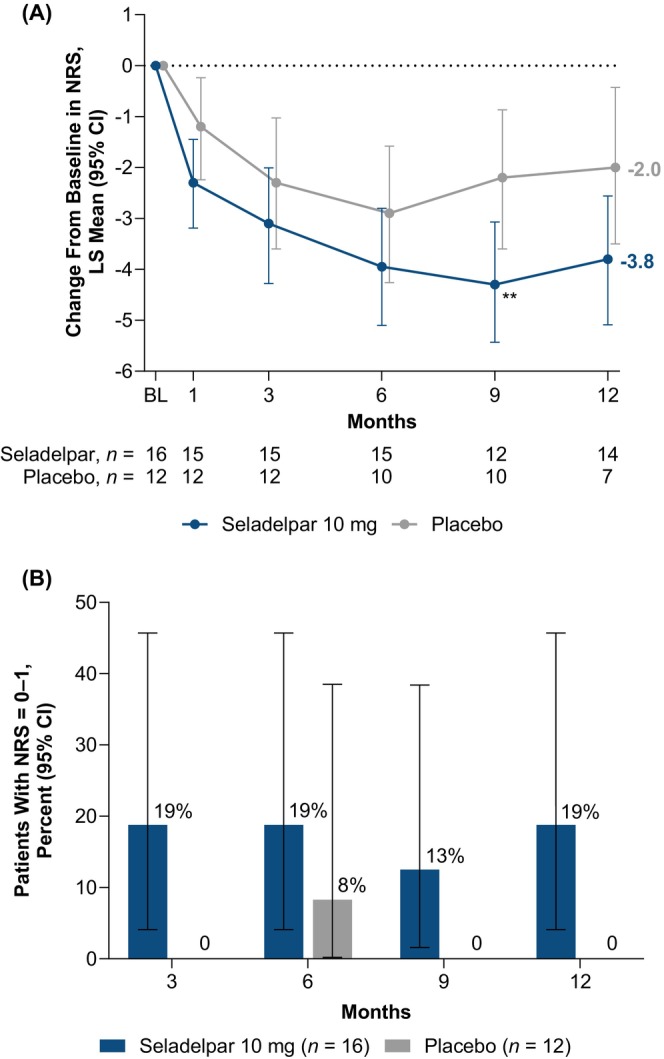
Change from BL in mean pruritus NRS over time (A) and percentage of patients with near resolution of itch (NRS = 0–1) (B) in patients with severe pruritus based on NRS ≥ 7 at BL. **In panel (A), *p* < 0.05 versus placebo. In panel (A), change from BL was estimated by a mixed‐effects model for repeated measures. *n* = the number of patients who had both a BL value and a value at that time point. In panel (B), patients with missing data at the specific time point were considered nonresponders. BL, baseline; LS, least‐square; NRS, numeric rating scale.

Among these patients, the reduction from baseline in NRS at 12 months was greater in those who were treated with seladelpar than in patients who received placebo (change from baseline, −3.8 points vs. −2.0 points; Figure 3A). At 12 months, 19% of patients who received seladelpar demonstrated near resolution of itch (NRS = 0–1) compared with 0% in the placebo group (Figure 3B).

Changes in 5‐D Itch domains and the sleep item are summarised in Figure S5. In general, numerical reductions in 5‐D Itch domains and the sleep item from baseline to 6 and 12 months were greater among patients who were treated with seladelpar than among those who received placebo.

**FIGURE 4:**
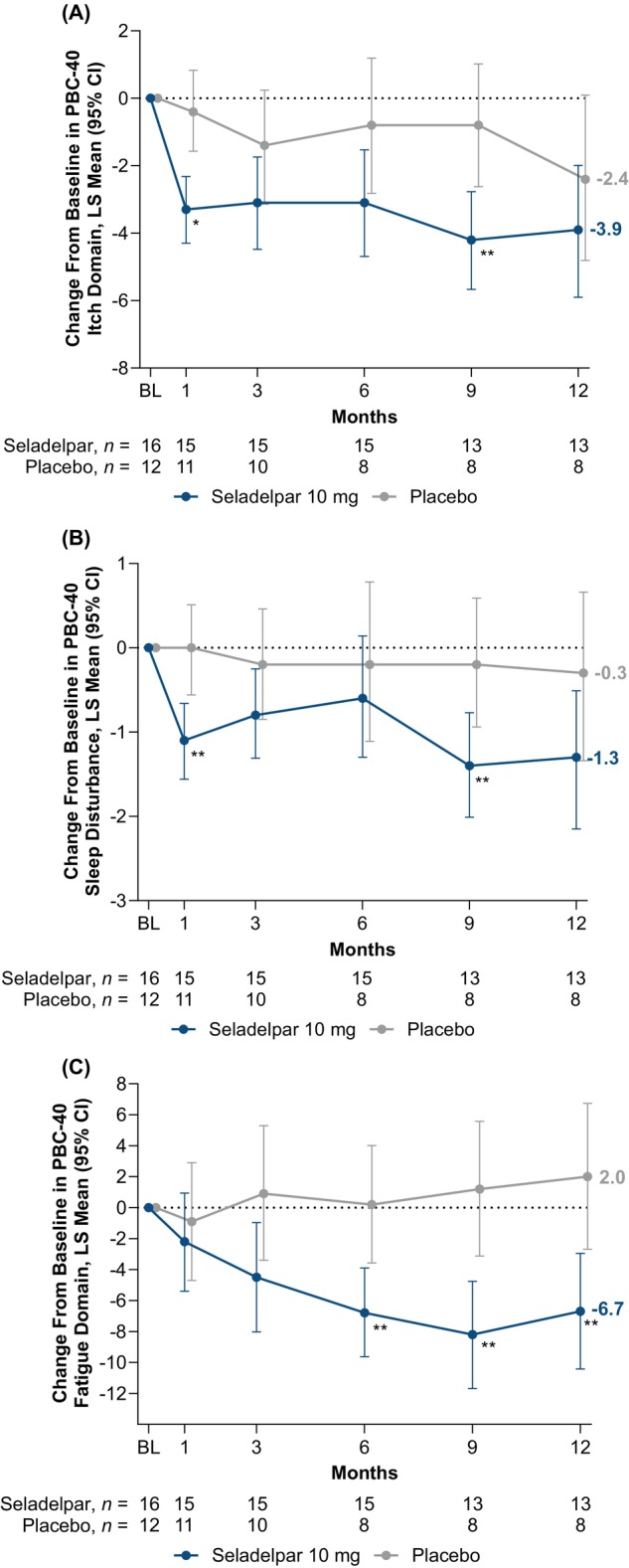
Change in PBC‐40 itch domain (A), sleep disturbance question (B) and fatigue domain (C) in patients with severe pruritus based on NRS ≥ 7 at BL. *p < 0.001 versus placebo. **p < 0.05 versus placebo. Change from BL was estimated by a mixed‐effects model for repeated measures. *n* = the number of patients who had both a BL value and a value at that time point. In panel (A), PBC‐40 Itch domain scores ranged from 0 to 15. In panel (B), PBC‐40 sleep disturbance question (within the itch domain) scores ranged from 0 to 5. In panel (C), PBC‐40 fatigue domain scores ranged from 11 to 55. BL, baseline; LS, least‐square; NRS, numeric rating scale; PBC, primary biliary cholangitis.

Changes from baseline in the PBC‐40 itch domain, sleep disturbance question and fatigue domain were analysed in patients with NRS ≥ 7 at baseline. A greater decrease in the LS mean PBC‐40 itch domain score was observed as early as after 1 month of seladelpar treatment versus placebo (*p* < 0.001), and greater reductions favouring seladelpar continued through 12 months (Figure 4A). Greater decreases in the PBC‐40 sleep disturbance question also occurred after 1 month of treatment with seladelpar versus placebo (*p* < 0.05), with an overall pattern of reduction through 12 months (Figure 4B). Lastly, a greater decrease in the LS mean PBC‐40 fatigue domain score was observed from 1 month through 12 months of treatment with seladelpar versus placebo (Figure 4C); the LS mean difference between arms was −8.7 points at 12 months, favouring seladelpar versus placebo (*p* < 0.05). Changes in all domains of the PBC‐40 and the sleep disturbance question at months 6 and 12 for patients with NRS ≥ 7 at baseline are shown in Figure S6.

### 3.3 Patients With Clinically Significant Pruritus (As Defined by PBC‐40 Itch ≥ 7) at Baseline

A total of 45 patients (35%) in the seladelpar group and 25 patients (38%) in the placebo group had a PBC‐40 itch domain score of ≥ 7 at baseline. Further demographic and baseline clinical characteristics for this group are reported in Table S5.

**FIGURE 5:**
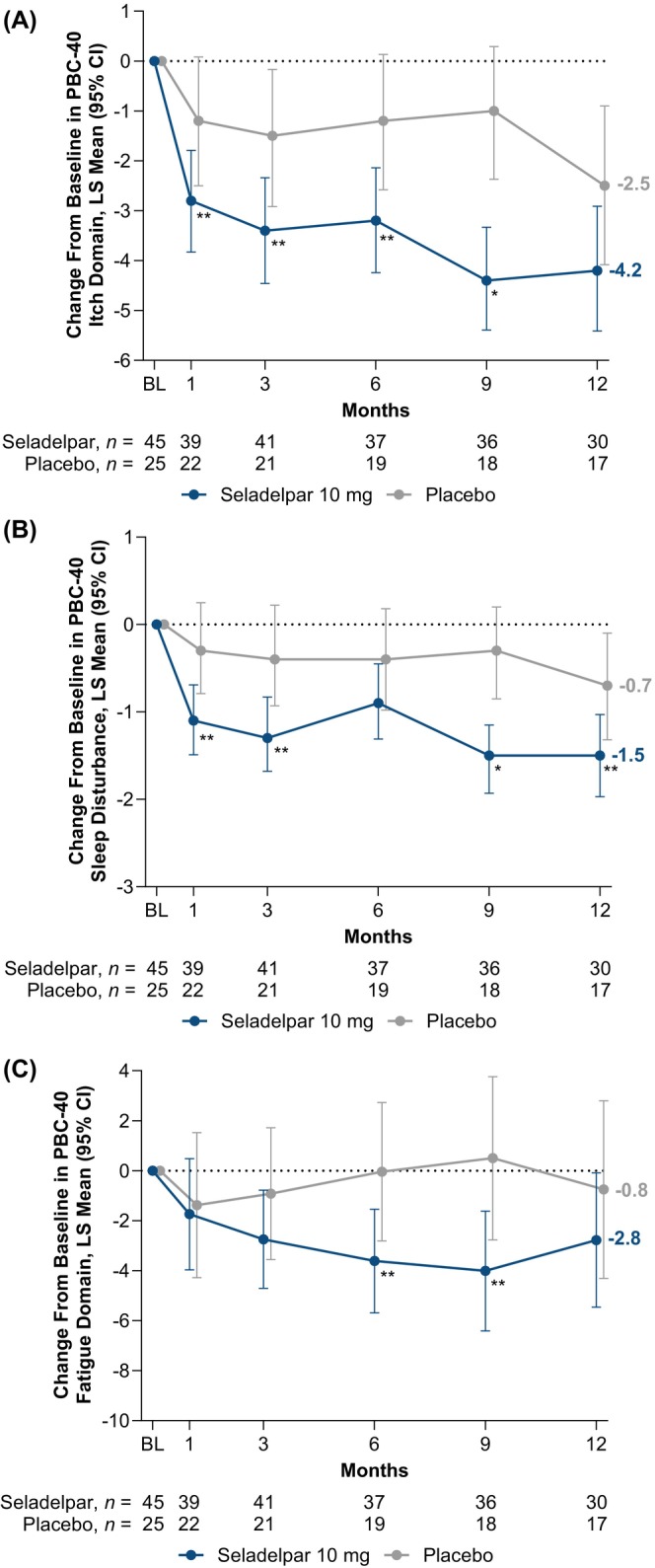
Change in PBC‐40 itch domain (A), sleep disturbance question (B) and fatigue domain (C) in patients with clinically significant pruritus based on PBC‐40 Itch ≥ 7 at BL. *p < 0.001 versus placebo. **p < 0.05 versus placebo. Change from BL was estimated by a mixed‐effects model for repeated measures. *n* = the number of patients who had both a BL value and a value at that time point. In panel (A), PBC‐40 itch domain scores ranged from 0 to 15. In panel (B), PBC‐40 sleep disturbance question (within the itch domain) scores ranged from 0 to 5. In panel (C), PBC‐40 fatigue domain scores ranged from 11 to 55. BL, baseline; LS, least‐square; PBC, primary biliary cholangitis.

Changes from baseline in the PBC‐40 itch domain, sleep disturbance question and fatigue domain were analysed for patients with PBC‐40 itch ≥ 7 at baseline. A greater decrease in the LS mean PBC‐40 itch domain score was observed as early as 1 month after initiating seladelpar treatment versus placebo, which was sustained for 9 months (*p* < 0.05); a similar numerical trend was observed at 12 months (Figure 5A). Improvements from PBC‐40 itch ≥ 7 to < 7 occurred in 40% and 20% of patients receiving seladelpar and placebo, respectively, at 12 months. A greater decrease in the LS mean PBC‐40 sleep disturbance question occurred as early as after 1 month of seladelpar treatment versus placebo (*p* < 0.05), and this difference was generally maintained through 12 months (*p* < 0.05) of treatment (Figure 5B). A greater decrease in the LS mean PBC‐40 fatigue domain score over time also occurred in patients with PBC‐40 itch ≥ 7 at baseline who were treated with seladelpar versus placebo (Figure 5C). Changes in all domains of the PBC‐40 and the sleep disturbance question at months 6 and 12 for patients with PBC‐40 itch ≥ 7 at baseline are shown in Figure S7.

### 3.4 Overall Population and Patients With No or Near No Itch (NRS = 0–1) at Baseline

**FIGURE 6:**
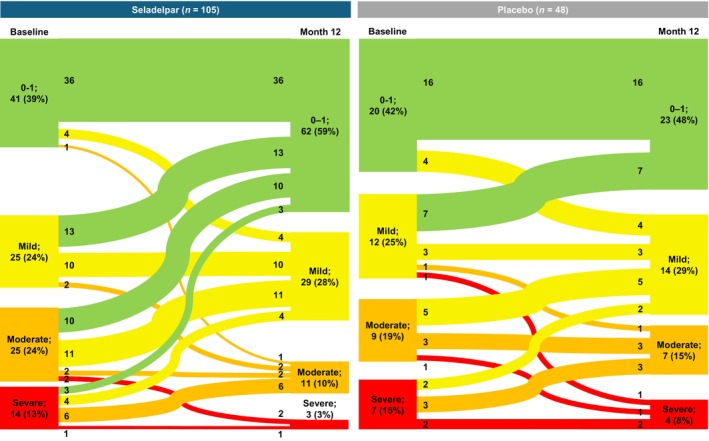
Sankey plot of BL and month 12 NRS categories for seladelpar and placebo in overall study population. A colour‐blind accessible version of this figure is available in Figure S10. Categories were defined as: No or near no itch (NRS = 0–1), mild (NRS > 1 to < 4), moderate (NRS 4 to < 7), or severe itch (NRS ≥ 7). Includes patients with data available at both BL and 12 months. BL, baseline; NRS, numeric rating scale.

Shifts from baseline to 12 months in pruritus NRS scores are presented in a Sankey plot (Figure 6). There was a pattern of improvement with more shifts to mild and minimal itching observed with seladelpar relative to placebo. Changes in pruritus medications were evaluated and were not considered to play a role, with three patients (2%) in the seladelpar group and four patients (6%) in the placebo group starting an antipruritic medication during the 12‐month study. A responder analysis assessed patients with no or near no itch at baseline. At baseline, 49 patients (38%) in the seladelpar group and 25 patients (38%) in the placebo group had no or near no itch (NRS = 0–1). Of these patients, 6% receiving seladelpar and 12% receiving placebo had an NRS score ≥ 2 at 12 months.

## 4 Safety

Overall, the proportions of patients who experienced AEs were similar in the seladelpar and placebo groups regardless of baseline itch severity (Table 2). The most common AEs by preferred term were similar to those observed in the primary analysis (Table S6) [12]. Pruritus reported as an AE was more common in patients receiving placebo (13%) than seladelpar (4%) in the NRS ≥ 4 population as well as in the NRS < 4 population (17% vs. 5%); none of these pruritus AEs were serious or led to treatment discontinuation. All pruritus AEs were grades 1 or 2 in severity, with the exception of one grade 3 event in the placebo arm in a patient with NRS ≥ 4 at baseline. The grade 3 event was treated with antihistamines and a corticosteroid. Among patients with NRS ≥ 4 at baseline, three patients (6%) receiving seladelpar and two patients (9%) receiving placebo experienced AEs leading to treatment discontinuation. Few AEs leading to treatment discontinuation occurred in the NRS < 4 group (1% [1/79], seladelpar; 2% [1/42], placebo).

**TABLE 2:** Overall safety by baseline pruritus NRS score (NRS < 4 and NRS ≥ 4) and treatment arm.

Patient incidence, *n* (%)	Patients with NRS < 4 at baseline		Patients with NRS ≥ 4 at baseline	
	Seladelpar (*n* = 79)	Placebo (*n* = 42)	Seladelpar (*n* = 49)	Placebo (*n* = 23)
Any AE	68 (86)	34 (81)	43 (88)	21 (91)
Grade ≥ 3 AEs (per CTCAE version 5.0)	9 (11)	2 (5)	5 (10)	3 (13)
SAEs	5 (6)	3 (7)	4 (8)	1 (4)
Treatment‐related SAEs	0	0	0	0
AEs leading to treatment discontinuation	1 (1)	1 (2)	3 (6)	2 (9)
AEs leading to death	0	0	0	0

*Note:* All AEs listed are treatment emergent unless otherwise stated.

Abbreviations: AE, adverse event; CTCAE, Common Terminology Criteria for Adverse Events; NRS, numeric rating scale; SAE, serious adverse event.

## 5 Discussion

These post hoc analyses further characterise the effect of seladelpar on pruritus observed in the pivotal, Phase 3 RESPONSE trial, in which seladelpar significantly reduced pruritus in patients with moderate to severe itch at baseline [12]. Here, we report a consistent pattern of improvement in pruritus, pruritus‐related sleep disturbance and measures of fatigue with seladelpar among patients with different baseline itch levels in the RESPONSE trial.

In a measure of benefit relevant to patients, seladelpar–treated patients with moderate to severe pruritus at baseline moved from moderate to mild itch based on group mean NRS values, while placebo–treated patients remained in the moderate range. At an individual patient level for patients with moderate to severe pruritus, a ≥ 3‐point decline in the NRS has been established to be clinically meaningful [16]. Although the study was not powered to assess a treatment difference in patients achieving this magnitude of decline, numerically more seladelpar–treated patients met this threshold. In addition, more patients receiving seladelpar experienced near resolution of itch compared with placebo. Results from the 5‐D Itch scale and the PBC‐40 itch domain support the NRS findings.

Duration and distribution of itch, which are also relevant to patients' experiences, were reduced with seladelpar. The 5‐D Itch body map suggests a reduction in extremity itch over time with seladelpar versus placebo. As PBC‐related pruritus usually affects the extremities, especially the palms and soles [6], these results may be considered clinically meaningful.

Fatigue poses a significant burden to PBC patients yet remains poorly understood. Here, we show that improvements in itch‐related sleep disturbance and measures of fatigue based on the 5‐D Itch and PBC‐40 scales in the NRS ≥ 4 population favoured seladelpar at months 6 and 12. Although small in number, patients with severe itching (NRS ≥ 7) at baseline demonstrated reductions in pruritus via the NRS and decreases in itch‐related sleep disturbance and measures of fatigue per the 5‐D Itch and PBC‐40 scales with seladelpar versus placebo. These results suggest that an effect on fatigue may be easier to detect among patients who are more symptomatic at baseline. These effects were generally consistent among patients with clinically significant itch at baseline (PBC‐40 itch ≥ 7). A moderate correlation between change in PBC‐40 itch and fatigue domain scores among patients with moderate to severe pruritus at baseline suggests that improvement in pruritus may be associated with improvement in fatigue, but also likely reflects the multifactorial nature of fatigue.

Of note, confidence intervals for several domains were wide or crossed zero, particularly for fatigue, sleep and some of the other quality of life domains (e.g., cognitive, emotional and social). This likely reflects variability in patient response and heterogeneity in factors contributing to these aspects of quality of life.

An additional consideration for patients and providers is mitigation of the onset of pruritus. The Sankey plot shows that seladelpar treatment tended to maintain patients without itching (no itch or near no itch) in that category while shifting other patients with higher baseline itch toward more mild itch categories. Safety findings further support this trend, with AEs of pruritus being more common with placebo compared to seladelpar in patients regardless of baseline pruritus status.

The findings presented here are consistent with earlier placebo‐controlled data from the ENHANCE study, in which seladelpar significantly reduced pruritus compared with placebo at 3 months [23]. Seladelpar has also been shown to decrease serum bile acids and the pruritogenic cytokine IL‐31, both of which have been implicated in the pathophysiology of cholestatic itch [24], supporting the clinical observations reported here.

Fatigue is a more challenging endpoint to measure due to many potential aetiologies and the impact of comorbidities [3, 5]. An earlier study reported a benefit of seladelpar on measures of fatigue in an open‐label setting [18]. However, placebo‐controlled data should remain the gold standard for assessing fatigue, in order to identify treatments with confirmed effects. Importantly, this analysis shows seladelpar to be the first second‐line therapy to demonstrate a significant difference in measures of fatigue versus placebo, albeit in a small number of patients with severe itch at baseline.

Overall, these results suggest seladelpar may offer an important new therapeutic option to address symptom burden and cholestasis. Historically, no approved treatments for PBC also improved pruritus [25]. First‐line UDCA does not relieve PBC‐related pruritus [25], and second‐line obeticholic acid has been known to cause pruritus [26]. Bile acid sequestrants (e.g., cholestyramine), rifampicin, opiate antagonists, selective serotonin reuptake inhibitors, gabapentin and antihistamines are sometimes used to treat pruritus, but they do not treat underlying PBC [1]. Further, they can increase pill burden, are supported by limited evidence, can come with safety concerns and are generally used off label [1].

Some data suggest fibrates benefit cholestatic pruritus [27, 28]. In the FITCH trial, 45% of patients with PBC or primary sclerosing cholangitis receiving bezafibrate demonstrated a ≥ 50% reduction in pruritus by visual analogue scale after 21 days of treatment compared with 11% of patients receiving placebo (*p* = 0.003) [28]. In the 24‐month BEZURSO study, however, bezafibrate did not significantly improve pruritus, but the population had relatively mild itch at baseline [27]. The PPAR agonist elafibranor demonstrated a trend toward improvement in pruritus based on the 5‐D Itch and PBC‐40 scales in the Phase 3 ELATIVE trial, although there was no significant difference in the key secondary endpoint of change on the Worst Itch NRS between elafibranor and placebo [29].

There are several limitations to the post hoc analyses presented here. The analyses did not control for multiple comparisons, and some of the analysis populations had small sample sizes. Protocol noncompliance with completion of questionnaires at later time points contributed to missing data, which further limited statistical power of the analyses. The data set for the evaluation of fatigue and other more subjective aspects of quality of life was limited to the predetermined questionnaires. Other factors that could contribute to changes in fatigue or quality of life were not evaluated (e.g., depression, thyroid disease, physical activity). Additional efforts to characterise mechanisms of fatigue and potential treatments, including a potential role for seladelpar to address this important symptom, are needed. Lastly, the analyses here focused on a 1‐year treatment period. Longer‐term data are needed to further characterise the effects of seladelpar on quality of life among patients with PBC over time.

## 6 Conclusions

Cholestatic pruritus is a prominent symptom among patients with PBC with limited treatment options. Fatigue, which is multifactorial in nature, also imposes a substantial burden on patients. In the RESPONSE trial, seladelpar led to meaningful reductions in itch severity and improved distribution of itch in patients with moderate to severe pruritus at baseline. Itch‐related sleep disturbance and measures of fatigue were also improved in patients with PBC and pruritus, while patients without itching at baseline tended to stay free from itching with seladelpar. In conclusion, in addition to an established anticholestatic effect, these data suggest a clinically relevant improvement in pruritus and other quality of life benefits from seladelpar in patients with PBC and pruritus.

## Author Contributions

The guarantor of the article is D.B.C. Conceptualisation: C.L., S.C., D.B.C., A.E.K. Data curation: All authors. Formal analysis: C.L., J.L., S.C., D.B.C., A.E.K. Funding acquisition: N/A. Investigation: All authors. Methodology: C.L., J.L., S.C., D.B.C., A.E.K. Project administration: All authors. Resources: All authors. Software: J.L. Supervision: C.L., S.C., D.B.C., A.E.K. Validation: J.L. Visualisation: C.L., J.L., S.C., D.B.C., A.E.K. Writing – original draft: C.L., J.L., S.C., D.B.C., A.E.K. Writing – review and editing: All authors.

## Funding

This work was supported by Gilead Sciences Inc.

## Conflicts of Interest

This study was funded by Gilead Sciences Inc. Medical writing and editorial support were provided by Ellie Manca, MPH, of Red Nucleus and were funded by Gilead Sciences Inc. C.L. reports receiving research grants paid to her institution from Calliditas Therapeutics; CymaBay Therapeutics; Escient Pharmaceuticals; EWR; Gilead Sciences Inc.; GSK; Intercept Pharmaceuticals; Ipsen; Kowa; Mirum Pharma; Target; and Zydus Pharmaceuticals. D.E.J.J. reports receiving consulting fees for CymaBay Therapeutics, Ipsen, Kowa and Umecrine, grants from Intercept, and participation on a speakers' bureau for Falk, GlaxoSmithKline, Intercept and Ipsen. M.J.M. reports receiving grants or contracts from CymaBay Therapeutics; Genfit; Gilead Sciences Inc.; GSK; Ipsen; and Mirum Pharma; consulting fees from CymaBay Therapeutics, GSK, Intra‐Sana Laboratories, Ipsen, Ironwood Pharmaceuticals, Mallinckrodt Pharmaceuticals and Mirum Pharma; and support for attending meetings and/or travel from CymaBay Therapeutics, GSK, Ipsen and Mallinckrodt Pharmaceuticals. C.L.B. reports receiving grants or contracts to his institution from Boston Scientific; Bristol Myers Squibb; Calliditas Therapeutics; Cara Therapeutics; Chemomab; COUR Pharmaceuticals; CymaBay Pharmaceuticals; Gilead Sciences Inc.; GSK; and Hanmi Pharmaceuticals; and consulting fees from Alnylam Pharmaceuticals; Chemomab; CymaBay Therapeutics; Gilead Sciences Inc.; GSK; Ipsen; and NGM Bio. K.V.K. reports receiving grants or contracts from 89Bio; Boston Pharmaceuticals; Corcept Therapeutics; CymaBay Therapeutics; Genfit; Gilead Sciences Inc.; GSK; Hanmi Pharmaceuticals; Intercept Pharmaceuticals; Ipsen; Janssen; Madrigal Pharmaceuticals; Mirum Pharma; Novo Nordisk; NGM Bio; Pfizer; Pliant Therapeutics; Terns Pharmaceuticals; Viking Therapeutics; and Zydus Pharmaceuticals; royalties or licences from UpToDate; consulting fees from 89Bio; CymaBay Therapeutics; Genfit; Gilead Sciences Inc.; GSK; HighTide Biopharma; Inipharm; Intercept Pharmaceuticals; Ipsen; Madrigal Pharmaceuticals; Mirum Pharma; NGM Bio; and Zydus Pharmaceuticals; payment or honoraria for lectures, presentations, speakers bureaus, manuscript writing, or educational events from AbbVie; Gilead Sciences Inc.; and Intercept Pharmaceuticals; participation on a data safety monitoring board or advisory board with CTI and Medpace; and stock or stock options with Inipharm. G.M.H. reports having consulted for Advanz Pharma; CymaBay Therapeutics; Falk; Gilead Sciences Inc.; GSK; Intercept Pharmaceuticals; Ipsen; Kowa; Mirum Pharma; and Pliant Therapeutics. J.L., C.B., S.C. and D.B.C. are employees of Gilead Sciences Inc., and may own stock in Gilead Sciences Inc. A.E.K. reports receiving grants or contracts from Gilead Sciences Inc., Intercept Pharmaceuticals and Roche; consulting fees from AbbVie; Advanz Pharma; Alentis Therapeutics; Alfasigma; Astellas; AstraZeneca; Attovia Therapeutics; Avior Bio; Bayer; Bristol Myers Squibb; Böhringer‐Ingelheim; CymaBay Therapeutics; Gilead Sciences Inc.; GSK; Guidepoint; Intercept Pharmaceuticals; Ipsen; Merck Sharp & Dohme; Mirum Pharma; Novo Nordisk; Rectify Pharma; Roche; and Takeda; payment or honoraria for lectures, presentations, speakers bureaus, manuscript writing, or educational events from AbbVie; Advanz Pharma; AOP Orphan Pharmaceuticals; Bayer; Bristol Myers Squibb; CymaBay Therapeutics; Falk; Gilead Sciences Inc.; GSK; Intercept Pharmaceuticals; Ipsen; Johnson & Johnson; Medscape; Merck Sharp & Dohme; Mirum Pharma; NewBridge Pharmaceuticals; Novartis; Roche; Vertex Pharmaceuticals; and Viofor; support for attending meetings and/or travel from Gilead Sciences Inc.; participation on a data safety monitoring board with AbbVie; Advanz Pharma; Alentis Therapeutics; Alfasigma; AstraZeneca; Avior Bio; Bayer; Bristol Myers Squibb; CymaBay Therapeutics; Escient Pharmaceuticals; Falk; Gilead Sciences Inc.; GSK; Guidepoint; Intercept Pharmaceuticals; Ipsen; Merck Sharp & Dohme; Mirum Pharma; Novo Nordisk; Roche; and Takeda; and a leadership or fiduciary role in other board, society, committee, or advocacy groups (paid or unpaid) with PBC Foundation, Swiss Association for the Study of the Liver (SASL), Swiss Gastroenterology Society (SGG), Swiss Hepa and Swiss Transplant Society (STS).

## Supporting information


**Table S1:** 5‐D Itch scale information.
**Table S2:** PBC‐40 scale information.
**Table S3:** Summary of health‐related quality of life outcomes reported by analysis population.
**Table S4:** Demographics and baseline clinical characteristics in patients with NRS ≥ 7 at BL.
**Table S5:** Demographics and baseline clinical characteristics in patients with PBC‐40 itch ≥ 7 at BL.
**Table S6:** Most common adverse events (≥ 5% in the seladelpar arms) by preferred term by BL pruritus NRS score (NRS < 4 and NRS ≥ 4) and treatment arm.
**Figure S1:** RESPONSE study design and schedule of PRO assessments.
**Figure S2:** 5‐D Itch changes in all domains and the sleep disturbance item at months 6 and 12 in patients with moderate to severe pruritus based on NRS ≥ 4 at baseline.
**Figure S3:** PBC‐40 changes in all domains and the sleep disturbance question at months 6 and 12 in patients with moderate to severe pruritus based on NRS ≥ 4 at baseline.
**Figure S4:** Spearman correlations of the change in PBC‐40 itch and fatigue domain scores at months 6 and 12 in patients with moderate to severe pruritus based on NRS ≥ 4 at baseline.
**Figure S5:** 5‐D Itch changes in all domains and the sleep disturbance item at months 6 and 12 in patients with severe pruritus based on NRS ≥ 7 at baseline.
**Figure S6:** PBC‐40 changes in all domains and the sleep disturbance question at months 6 and 12 in patients with severe pruritus based on NRS ≥ 7 at baseline.
**Figure S7:** PBC‐40 changes in all domains and the sleep disturbance question at months 6 and 12 in patients with clinically significant pruritus based on PBC‐40 itch ≥ 7 at baseline.
**Figure S8:** Mean pruritus NRS over time in patients with moderate to severe pruritus based on NRS ≥ 4 at BL (colour‐blind accessible).
**Figure S9:** Daily duration of itch (a) and body regions with pruritus (b) over time based on 5‐D Itch in patients with moderate to severe pruritus based on NRS ≥ 4 at baseline (colour‐blind accessible).
**Figure S10:** Sankey plot of baseline and month 12 NRS categories for seladelpar and placebo in overall study population (colour‐blind accessible).

## Data Availability

Gilead Sciences shares anonymised individual patient data upon request or as required by law or regulation with qualified external researchers based on submitted curriculum vitae and reflecting non conflict of interest. The request proposal must also include a statistician. Approval of such requests is at Gilead Sciences' discretion and is dependent on the nature of the request, the merit of the research proposed, the availability of the data, and the intended use of the data. Data requests should be sent to datarequest@gilead.com.

## References

[1] EASL Clinical Practice Guidelines , “The Diagnosis and Management of Patients With Primary Biliary Cholangitis,” Journal of Hepatology 67, no. 1 (2017): 145–172, 10.1016/j.jhep.2017.03.022. 28427765

[2] K. D. Lindor , C. L. Bowlus , J. Boyer , C. Levy , and M. Mayo , “Primary Biliary Cholangitis: 2018 Practice Guidance From the American Association for the Study of Liver Diseases,” Hepatology 69, no. 1 (2019): 394–419, 10.1002/hep.30145. 30070375

[3] F. Q. Onofrio , G. M. Hirschfield , and A. F. Gulamhusein , “A Practical Review of Primary Biliary Cholangitis for the Gastroenterologist,” Gastroenterology Hepatology (New York) 15, no. 3 (2019): 145–154. PMC6495411 31061656

[4] B. Schnabl , “PPAR Agonists in Primary Biliary Cholangitis,” New England Journal of Medicine 390, no. 9 (2024): 855–858, 10.1056/NEJMe2313802. 38381666

[5] J. Trivella , B. V. John , and C. Levy , “Primary Biliary Cholangitis: Epidemiology, Prognosis, and Treatment,” Hepatology Communications 7, no. 6 (2023): e0179, 10.1097/hc9.0000000000000179. 37267215 PMC10241503

[6] M. M. Düll and A. E. Kremer , “Evaluation and Management of Pruritus in Primary Biliary Cholangitis,” Clinics in Liver Disease 26, no. 4 (2022): 727–745, 10.1016/j.cld.2022.06.009. 36270726

[7] M. J. Mayo , E. Carey , H. T. Smith , et al., “Impact of Pruritus on Quality of Life and Current Treatment Patterns in Patients With Primary Biliary Cholangitis,” Digestive Diseases and Sciences 68, no. 3 (2023): 995–1005, 10.1007/s10620-022-07581-x. 35704252 PMC10406656

[8] H. T. Smith , S. Das , J. Fettiplace , et al., “Pervasive Role of Pruritus in Impaired Quality of Life in Patients With Primary Biliary Cholangitis: Data From the GLIMMER Study,” Hepatology Communications 9, no. 3 (2025): e0635, 10.1097/hc9.0000000000000635. 39969430 PMC11841849

[9] Livdelzi. Prescribing Information (Gilead Sciences Inc, 2024).

[10] Livdelzi. UK Summary of Product Characteristics (Gilead Sciences Inc., 2024).

[11] Lyvdelzi. EMA Prescribing Information (Gilead Sciences Inc, 2025).

[12] G. M. Hirschfield , C. L. Bowlus , M. J. Mayo , et al., “A Phase 3 Trial of Seladelpar in Primary Biliary Cholangitis,” New England Journal of Medicine 390, no. 9 (2024): 783–794, 10.1056/NEJMoa2312100. 38381664

[13] R. von Maltzahn , M. J. Mayo , H. T. Smith , et al., “Relationship Between Pruritus and Sleep in Participants With Primary Biliary Cholangitis in the Phase 2b GLIMMER Trial,” Journal of Patient‐Reported Outcomes 8, no. 1 (2024): 60, 10.1186/s41687-024-00722-y. 38862718 PMC11166618

[14] G. Yosipovitch , M. Reaney , V. Mastey , et al., “Peak Pruritus Numerical Rating Scale: Psychometric Validation and Responder Definition for Assessing Itch in Moderate‐to‐Severe Atopic Dermatitis,” British Journal of Dermatology 181, no. 4 (2019): 761–769, 10.1111/bjd.17744. 30729499 PMC6850643

[15] A. E. Kremer , D. Jones , A. M. Skalicky , et al., EASL 2025 (Poster THU‐277, 2025).

[16] C. Levy , A. E. Kremer , A. M. Skalicky , et al., EASL 2025 (Poster THU‐291, 2025).

[17] S. Elman , L. S. Hynan , V. Gabriel , and M. J. Mayo , “The 5‐D Itch Scale: A New Measure of Pruritus,” British Journal of Dermatology 162, no. 3 (2010): 587–593, 10.1111/j.1365-2133.2009.09586.x. 19995367 PMC2875190

[18] A. E. Kremer , M. J. Mayo , G. Hirschfield , et al., “Seladelpar Improved Measures of Pruritus, Sleep, and Fatigue and Decreased Serum Bile Acids in Patients With Primary Biliary Cholangitis,” Liver International 42, no. 1 (2022): 112–123, 10.1111/liv.15039. 34403559

[19] A. Jacoby , A. Rannard , D. Buck , et al., “Development, Validation, and Evaluation of the PBC‐40, a Disease Specific Health Related Quality of Life Measure for Primary Biliary Cirrhosis,” Gut 54, no. 11 (2005): 1622–1629, 10.1136/gut.2005.065862. 15961522 PMC1774759

[20] N. Al‐Harthy , T. Kumagi , C. Coltescu , and G. M. Hirschfield , “The Specificity of Fatigue in Primary Biliary Cirrhosis: Evaluation of a Large Clinic Practice,” Hepatology 52, no. 2 (2010): 562–570, 10.1002/hep.23683. 20683955

[21] D. Jones , M. Carbone , P. Invernizzi , et al., “Impact of Setanaxib on Quality of Life Outcomes in Primary Biliary Cholangitis in a Phase 2 Randomized Controlled Trial,” Hepatology Communications 7, no. 3 (2023): e0057, 10.1097/hc9.0000000000000057. 36809195 PMC9949832

[22] G. R. Marczyk , D. DeMatteo , and D. Festinger , Essentials of Research Design and Methodology (John Wiley & Sons, 2010).

[23] G. M. Hirschfield , M. L. Shiffman , A. Gulamhusein , et al., “Seladelpar Efficacy and Safety at 3 Months in Patients With Primary Biliary Cholangitis: ENHANCE, a Phase 3, Randomized, Placebo‐Controlled Study,” Hepatology 78, no. 2 (2023): 397–415, 10.1097/hep.0000000000000395. 37386786 PMC10344437

[24] A. E. Kremer , M. J. Mayo , G. M. Hirschfield , et al., “Seladelpar Treatment Reduces IL‐31 and Pruritus in Patients With Primary Biliary Cholangitis,” Hepatology 80, no. 1 (2024): 27–37, 10.1097/hep.0000000000000728. 38117036 PMC11191048

[25] C. Levy , M. Manns , and G. Hirschfield , “New Treatment Paradigms in Primary Biliary Cholangitis,” Clinical Gastroenterology and Hepatology 21, no. 8 (2023): 2076–2087, 10.1016/j.cgh.2023.02.005. 36809835

[26] F. Nevens , P. Andreone , G. Mazzella , et al., “A Placebo‐Controlled Trial of Obeticholic Acid in Primary Biliary Cholangitis,” New England Journal of Medicine 375, no. 7 (2016): 631–643, 10.1056/NEJMoa1509840. 27532829

[27] C. Corpechot , O. Chazouillères , A. Rousseau , et al., “A Placebo‐Controlled Trial of Bezafibrate in Primary Biliary Cholangitis,” New England Journal of Medicine 378, no. 23 (2018): 2171–2181, 10.1056/NEJMoa1714519. 29874528

[28] E. de Vries , R. Bolier , J. Goet , et al., “Fibrates for Itch (FITCH) in Fibrosing Cholangiopathies: A Double‐Blind, Randomized, Placebo‐Controlled Trial,” Gastroenterology 160, no. 3 (2021): 734–743.e6, 10.1053/j.gastro.2020.10.001. 33031833

[29] K. V. Kowdley , C. L. Bowlus , C. Levy , et al., “Efficacy and Safety of Elafibranor in Primary Biliary Cholangitis,” New England Journal of Medicine 390, no. 9 (2024): 795–805, 10.1056/NEJMoa2306185. 37962077

