# Supplementary material for: Seladelpar Improved Itch, Itch‐Related Sleep Disturbance and Measures of Fatigue in Patients With Primary Biliary Cholangitis and Pruritus in the Phase 3 RESPONSE Trial
Source: Aliment Pharmacol Ther. 2026 Apr 3;64(1):23–35. doi: 10.1111/apt.70630 (PMC13251588; doi:10.1111/apt.70630)
Supplement: Supplementary file 1 — Table S1: 5‐D Itch scale information. Table S2: PBC‐40 scale information. Table S3: Summary of health‐related quality of life outcomes reported by analysis population. Table S4: Demographics and baseline clinical characteristics in patients with NRS ≥ 7 at BL. Table S5: Demographics and baseline clinical characteristics in patients with PBC‐40 itch ≥ 7 at BL. Table S6: Most common adverse events (≥ 5% in the seladelpar arms) by preferred term by BL pruritus NRS score (NRS < 4 and NRS ≥ 4) and treatment arm. Figure S1: RESPONSE study design and schedule of PRO assessments. Figure S2: 5‐D Itch changes in all domains and the sleep disturbance item at months 6 and 12 in patients with moderate to severe pruritus based on NRS ≥ 4 at baseline. Figure S3: PBC‐40 changes in all domains and the sleep disturbance question at months 6 and 12 in patients with moderate to severe pruritus based on NRS ≥ 4 at baseline. Figure S4: Spearman correlations of the change in PBC‐40 itch and fatigue domain scores at months 6 and 12 in patients with moderate to severe pruritus based on NRS ≥ 4 at baseline. Figure S5: 5‐D Itch changes in all domains and the sleep disturbance item at months 6 and 12 in patients with severe pruritus based on NRS ≥ 7 at baseline. Figure S6: PBC‐40 changes in all domains and the sleep disturbance question at months 6 and 12 in patients with severe pruritus based on NRS ≥ 7 at baseline. Figure S7: PBC‐40 changes in all domains and the sleep disturbance question at months 6 and 12 in patients with clinically significant pruritus based on PBC‐40 itch ≥ 7 at baseline. Figure S8: Mean pruritus NRS over time in patients with moderate to severe pruritus based on NRS ≥ 4 at BL (colour‐blind accessible). Figure S9: Daily duration of itch (a) and body regions with pruritus (b) over time based on 5‐D Itch in patients with moderate to severe pruritus based on NRS ≥ 4 at baseline (colour‐blind accessible). Figure S10: Sankey plot of baseline and month 12 NRS categories for s [file APT-64-23-s001.zip › apt70630-sup-0001-Supinfo01.docx]

# Supplemental Material

## **Supplemental Methods**

For “Any pruritus medication at baseline,” only antipruritic medications started on or prior to baseline and ended on or after baseline were included. Antipruritic medications included in the search strategy were cholestyramine, rifampicin, naltrexone, naloxone, nalmefene, sertraline, gabapentin, nalfurafine, colesevelam, colestipol, sertraline hydrochloride, naltrexone hydrochloride, and naloxone hydrochloride.

### ***Supplemental Table 1.*** *5-D Itch scale information*^1,2^

| **5-D Itch Domain** | **Description^†^** | **Answer Choices** | **Point Equivalent** |
| --- | --- | --- | --- |
| Duration | Hours per day | <6; 6–12; 12–18; 18–23; All day | Answers correspond to a score of 1 to 5, with higher scores indicating greater impairment |
| Degree | Severity | Not present; Mild; Moderate; Severe; Unbearable |  |
| Direction | Improvement or worsening | Completely resolved; Much better but still present; Little bit better but still present; Unchanged; Getting worse |  |
| Disability | 4 items addressing impact of itch on  (1) sleep,  (2) leisure/social,  (3) housework/errands, and (4) work/school | (1) Sleep   - Never affects sleep - Occasionally delays falling asleep - Frequently delays falling asleep - Delays falling asleep and occasionally wakes me up at night - Delays falling asleep and frequently wakes me up at night   (2, 3, 4) Leisure/social, housework/errands, work/school   - Never affects this activity - Rarely affects this activity - Occasionally affects this activity - Frequently affects this activity - Always affects this activity |  |
| Distribution | 16 potential affected body regions (abdomen, back, buttocks, chest, contact with clothing, face, forearms, groin, head/scalp, lower legs, palms, soles, thighs, tops of feet/toes, tops of hands/fingers, upper arms) | Absent or present | The score for this domain is the total number of affected body parts sorted into 5 bins: 1 = sum of 0–2, 2 = sum of 3–5, 3 = sum of 6–10, 4 = sum of 11–13, 5 = sum of 14–16 |

**^†^**The recall period is over the past 2 weeks for all domains.

N/A, not applicable.

**The 5-D Itch Scale**


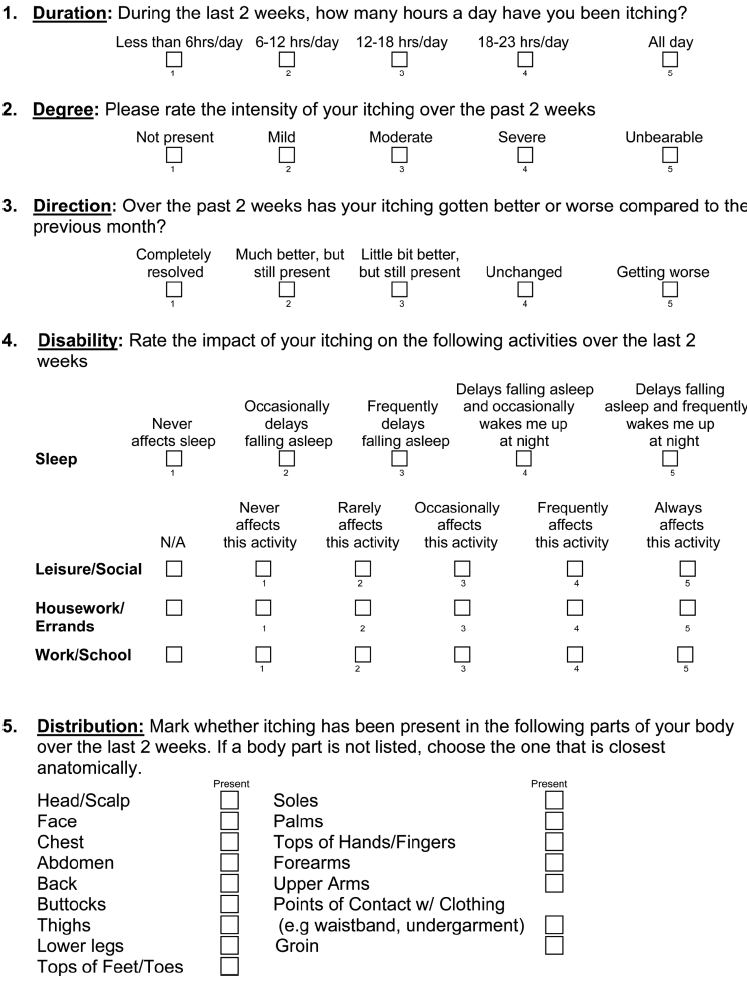


Elman S, et al. The 5-D itch scale: a new measure of pruritus. *Br J Dermatol.* 2010;162(3):587-93, with permission of Oxford University Press.^2^

### ***Supplemental Table 2.*** *PBC-40 scale information*^3,4^

| **PBC-40 Domain** | **Questions Within Each Domain** | **Answer Choices** | **Score Range (higher scores indicate greater impairment)** | **Recall Period** |
| --- | --- | --- | --- | --- |
| Itch | 3 | Did not apply/no itch; Never; Rarely; Sometimes; Most of the time; Always | 0 to 15 | Over past 4 weeks |
| Question for how itching disturbed sleep | N/A |  | 0 to 5 |  |
| Fatigue | 11 | Never; Rarely; Sometimes; Most of the time; Always | 11 to 55 |  |
| Social | 10 | Not at all; A little; Somewhat; Quite a bit; Very much | 8 to 50 | Overall/in general |
| Cognitive | 6 | Never; Rarely; Sometimes; Most of the time; Always | 6 to 30 | Over past 4 weeks |
| Emotional | 3 | Not at all; A little; Somewhat; Quite a bit; Very much | 3 to 15 | Overall/in general |
| Symptoms | 7 | Never; Rarely; Sometimes; Most of the time; Always | 6 to 35 | Over past 4 weeks |

N/A, not applicable; PBC, primary biliary cholangitis.

**The PBC-40 Questionnaire**


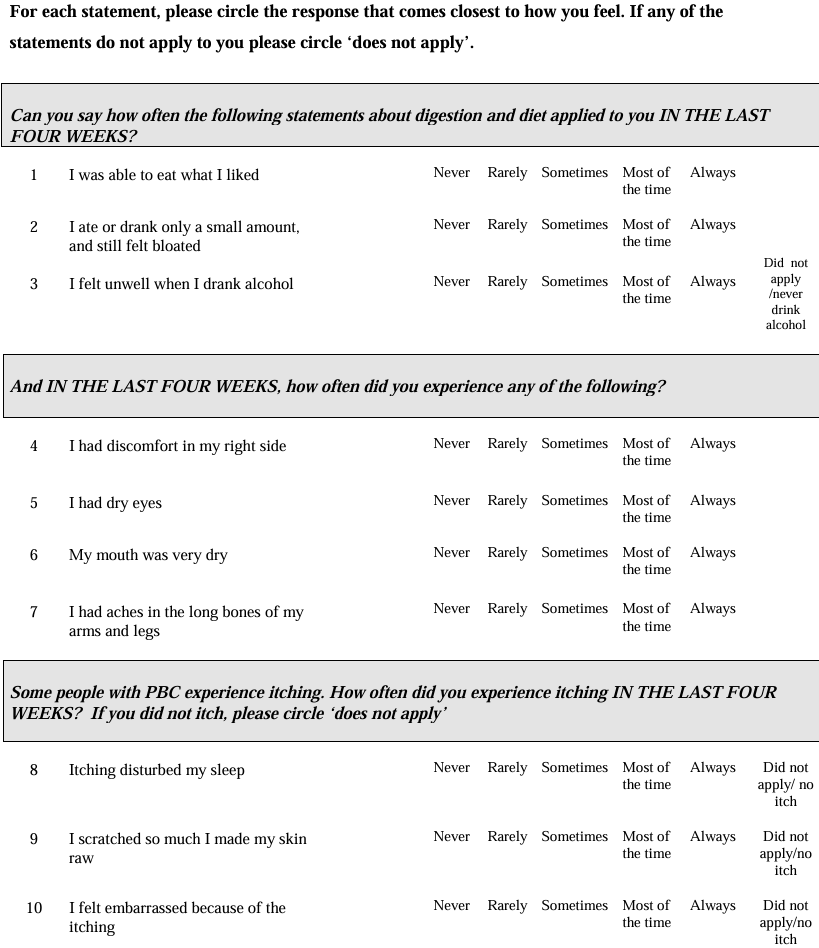


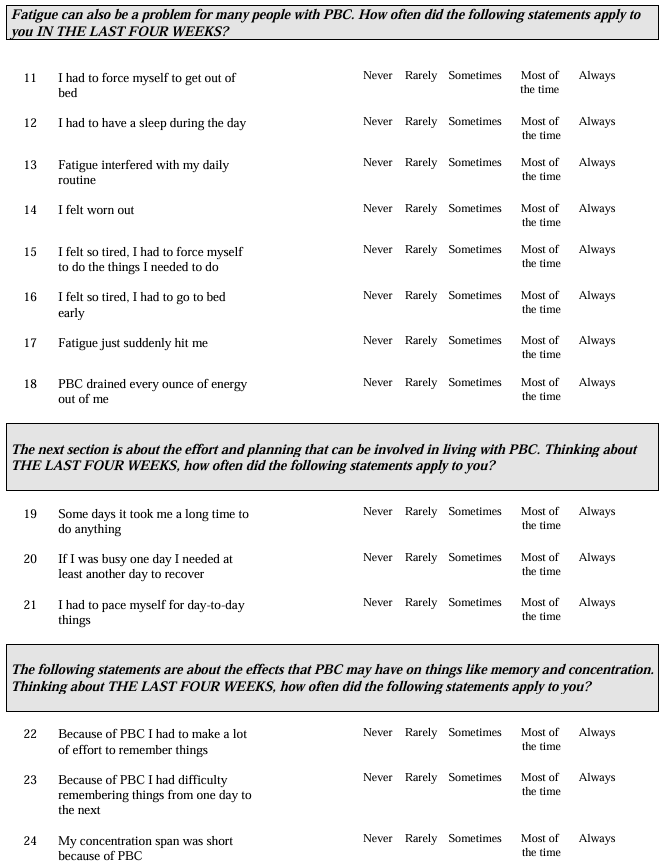


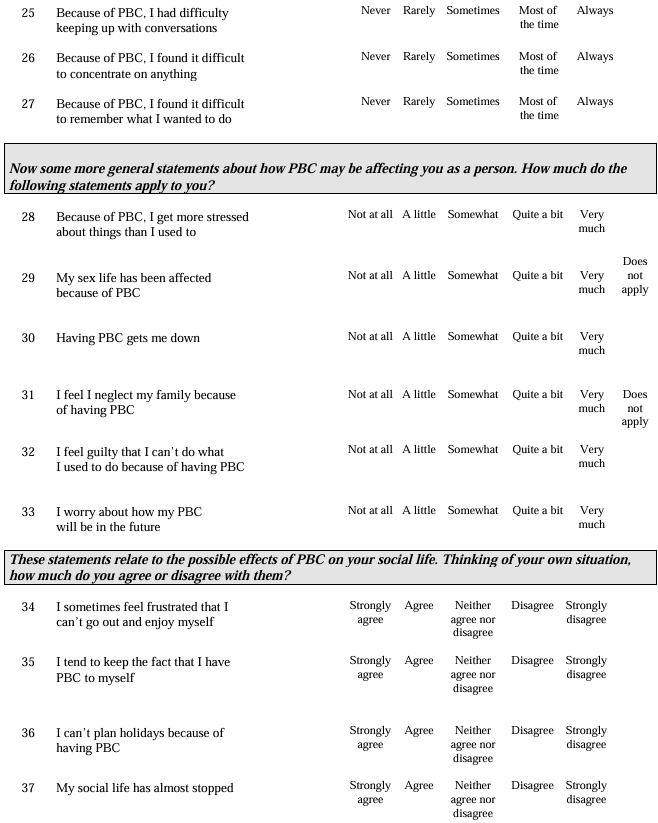


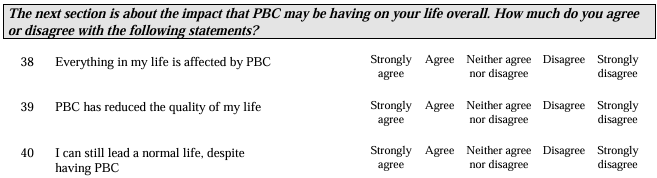


The PBC-40 questionnaire is available at <https://www.uk-pbc.com/resources/tools/pbc-40/>.

PBC, primary biliary cholangitis.

### ***Supplemental Table 3.*** *Summary of health-related quality of life outcomes reported by analysis population*

| **Analysis Name​** | **NRS ≥4​** | **NRS ≥7​** | **PBC-40 Itch ≥7​** | **NRS = 0–1 ​** | **Overall population** |
| --- | --- | --- | --- | --- | --- |
| **NRS mean change from baseline​** | † | X​ | ​ | ​ | † |
| **NRS mean values over time​** | X​ | ​ | ​ | ​ |  |
| **NRS 3- and 4-point improvement (6 and 12 months)​** | X​ | ​ | ​ | ​ |  |
| **NRS near resolution of itch (NRS = 0–1) over time** | X​ | X​ | ​ | ​ |  |
| **5-D Itch mean changes from baseline and mean values in total, all domains, and sleep ​over time** | † |  | ​ | ​ | † |
| **5-D Itch forest plot for all domains and sleep item (6 and 12 months)​** | X​ | X​ | ​ | ​ |  |
| **5-D Itch duration domain (6 and 12 months)** | X |  |  |  |  |
| **5-D Itch body map of distribution domain (6 and 12 months)​** | X​ | ​ | ​ | ​ |  |
| **PBC-40 mean changes from baseline and mean values in itch, fatigue, and sleep​ disturbance over time** | †**^‡^** | X​**^§^** | X**^§^** | ​ | †**^‡^** |
| **PBC-40 forest plot for all domains and sleep disturbance question (6 and 12 months)​** | X​ | X​ | X​ | ​ |  |
| **Shifts from PBC-40 itch ≥7 to <7​ (12 months)** | ​ | ​ | X​ | ​ |  |
| **Development of itch (NRS ≥2) (12 months)** | ​ | ​ | ​ | X​ |  |
| **Sankey plot of NRS category shifts (12 months)** |  |  |  |  | X |

A † represents data already published in the primary RESPONSE publication (Hirschfield, et al. *N Engl J Med.* 2024;390[9]:783-794). An X represents a new analysis reported in this manuscript.

**^‡^**Fatigue domain was not included in the primary RESPONSE publication. **^§^**Mean values over time are not reported in this manuscript (while mean changes from baseline are reported).

NRS, numeric rating scale; PBC, primary biliary cholangitis.

# Supplemental Results

## **Supplemental Tables**

### ***Supplemental Table 4.*** *Demographics and baseline clinical characteristics in patients with NRS ≥7 at BL*

| **Characteristic** | Seladelpar  (n = 16) | Placebo  (n = 12) |
| --- | --- | --- |
| **Age, years, mean (SD)** | 54 (10.7) | 56 (10.9) |
| **Age at PBC diagnosis, years, mean (SD)** | 47 (10.9) | 49 (11.0) |
| Diagnosed at <50 years, n (%) | 9 (56) | 5 (42) |
| Diagnosed at ≥50 years, n (%) | 7 (44) | 7 (58) |
| **Female, n (%)** | 15 (94) | 12 (100) |
| **Race, n (%)** |  |  |
| American Indian or Alaska Native | 2 (13) | 1 (8) |
| Asian | 0 | 0 |
| Black or African American | 0 | 0 |
| White | 14 (88) | 11 (92) |
| **Ethnicity, n (%)** |  |  |
| Hispanic or Latino | 8 (50) | 5 (42) |
| Not Hispanic or Latino | 8 (50) | 7 (58) |
| **Duration of PBC, years, mean (SD)** | 8 (4.4) | 8 (7.1) |
| **History of fatigue, n (%)** | 9 (56) | 7 (58) |
| **Prior use of OCA/fibrates, n (%)** | 6 (38) | 4 (33) |
| **Any pruritus medication at baseline,^†^ n (%)** | 3 (19) | 2 (17) |
| Cholestyramine | 1 (6) | 2 (17) |
| Rifampicin | 1 (6) | 0 |
| Gabapentin | 1 (6) | 0 |
| Sertraline | 2 (13) | 0 |
| **UDCA intolerant, n (%)** | 1 (6) | 1 (8) |
| **NRS score, mean (SD)** | 7.8 (0.6) | 7.8 (0.7) |
| **PBC-40 scores** |  |  |
| Itch domain score, mean (SD) | 10.6 (2.4) | 10.5 (2.0) |
| Sleep question score, mean (SD) | 3.8 (0.6) | 3.8 (0.8) |
| Fatigue domain score, mean (SD) | 31.3 (8.1) | 36.3 (6.7) |
| **Cirrhosis, n (%)** | 4 (25) | 2 (17) |
| **ALP,^‡^ U/L, mean (SD)** | 380 (164) | 331 (145) |
| **ALT,^§^ U/L, mean (SD)** | 57 (29) | 59 (28) |
| **Total bilirubin,^¶^ mg/dL, mean (SD)** | 0.92 (0.34) | 0.72 (0.34) |
| **GGT,^#^ U/L, mean (SD)** | 317 (214) | 404 (311) |

**^†^**Details regarding pruritus medications are expanded upon in the **Supplemental Methods**. **^‡^**The ULN is 116 U/L in men and women. **^§^**The ULN is 41 U/L in men and women. **^¶^**The ULN is 1.10 mg/dL in men and women. **^#^**The ULN is 52 U/L in men and 38 U/L in women.

ALP, alkaline phosphatase; ALT, alanine aminotransferase; GGT, gamma-glutamyl transferase; NRS, numeric rating scale; OCA, obeticholic acid; PBC, primary biliary cholangitis; UDCA, ursodeoxycholic acid; ULN, upper limit of normal.

### ***Supplemental Table 5.*** *Demographics and baseline clinical characteristics in patients with PBC-40 itch ≥7 at BL*

| **Characteristic** | Seladelpar  (n = 45) | Placebo  (n = 25) |
| --- | --- | --- |
| **Age, years, mean (SD)** | 52 (10.9) | 55 (11.1) |
| **Age at PBC diagnosis, years, mean (SD)** | 46 (10.9) | 47 (12.1) |
| Diagnosed at <50 years, n (%) | 26 (58) | 14 (56) |
| Diagnosed at ≥50 years, n (%) | 19 (42) | 11 (44) |
| **Female, n (%)** | 44 (98) | 24 (96) |
| **Race, n (%)** |  |  |
| American Indian or Alaska Native | 2 (4) | 1 (4) |
| Asian | 1 (2) | 1 (4) |
| Black or African American | 2 (4) | 0 |
| White | 39 (87) | 23 (92) |
| Missing | 1 (2) | 0 |
| **Ethnicity, n (%)** |  |  |
| Hispanic or Latino | 14 (31) | 10 (40) |
| Not Hispanic or Latino | 30 (67) | 15 (60) |
| Missing | 1 (2) | 0 |
| **Duration of PBC, years, mean (SD)** | 7 (5.1) | 9 (7.2) |
| **History of fatigue, n (%)** | 29 (64) | 16 (64) |
| **Prior use of OCA/fibrates, n (%)** | 11 (24) | 7 (28) |
| **Any pruritus medication at baseline,^†^ n (%)** | 11 (24) | 7 (28) |
| Cholestyramine | 5 (11) | 5 (20) |
| Colestipol | 0 | 1 (4) |
| Rifampicin | 3 (7) | 1 (4) |
| Gabapentin | 2 (4) | 0 |
| Sertraline | 3 (7) | 0 |
| **UDCA intolerant, n (%)** | 3 (7) | 1 (4) |
| **NRS score, mean (SD)** | 5.8 (1.8) | 5.9 (2.3) |
| **PBC-40 scores** |  |  |
| Itch domain score, mean (SD) | 9.6 (1.8) | 9.9 (2.0) |
| Sleep question score, mean (SD) | 3.5 (0.7) | 3.4 (0.9) |
| Fatigue domain score, mean (SD) | 32.4 (8.5) | 34.2 (8.6) |
| **Cirrhosis, n (%)** | 9 (20) | 4 (16) |
| **ALP,^‡^ U/L, mean (SD)** | 352 (147) | 315 (119) |
| **ALT,^§^ U/L, mean (SD)** | 54 (27) | 53 (24) |
| **Total bilirubin,^¶^ mg/dL, mean (SD)** | 0.84 (0.30) | 0.77 (0.37) |
| **GGT,^#^ U/L, mean (SD)** | 306 (273) | 312 (246) |

**^†^**Details regarding pruritus medications are expanded upon in the **Supplemental Methods**. **^‡^**The ULN is 116 U/L in men and women. **^§^**The ULN is 41 U/L in men and women. **^¶^**The ULN is 1.10 mg/dL in men and women. **^#^**The ULN is 52 U/L in men and 38 U/L in women.

ALP, alkaline phosphatase; ALT, alanine aminotransferase; GGT, gamma-glutamyl transferase; NRS, numeric rating scale; OCA, obeticholic acid; PBC, primary biliary cholangitis; UDCA, ursodeoxycholic acid; ULN, upper limit of normal.

### ***Supplemental Table 6.*** *Most common adverse events (≥5% in the seladelpar arms) by preferred term by BL pruritus NRS score (NRS <4 and NRS ≥4) and treatment arm*

| **Patient incidence, n (%)** | Patients with NRS <4 at baseline | | Patients with NRS ≥4 at baseline | |
| --- | --- | --- | --- | --- |
|  | Seladelpar (n = 79) | Placebo (n = 42) | Seladelpar (n = 49) | Placebo (n = 23) |
| **At least 1 AE** | 68 (86) | 34 (81) | 43 (88) | 21 (91) |
| COVID-19 | 12 (15) | 7 (17) | 11 (22) | 3 (13) |
| Abdominal distension | 7 (9) | 1 (2) | 1 (2) | 1 (4) |
| Arthralgia | 7 (9) | 1 (2) | 1 (2) | 3 (13) |
| Nausea | 6 (8) | 3 (7) | 2 (4) | 0 |
| Fatigue | 6 (8) | 1 (2) | 2 (4) | 3 (13) |
| Diarrhoea | 5 (6) | 3 (7) | 1 (2) | 0 |
| Anaemia | 5 (6) | 2 (5) | 1 (2) | 0 |
| Abdominal pain | 5 (6) | 1 (2) | 4 (8) | 0 |
| Pruritus | 4 (5) | 7 (17) | 2 (4) | 3 (13) |
| Nasopharyngitis | 4 (5) | 3 (7) | 3 (6) | 2 (9) |
| Headache | 4 (5) | 2 (5) | 6 (12) | 0 |
| Vitamin D deficiency | 4 (5) | 2 (5) | 2 (4) | 0 |
| Alopecia | 4 (5) | 1 (2) | 0 | 0 |
| Constipation | 4 (5) | 0 | 1 (2) | 3 (13) |
| Dyspepsia | 4 (5) | 0 | 1 (2) | 1 (4) |
| Pharyngitis | 0 | 2 (5) | 4 (8) | 3 (13) |
| Urinary tract infection | 1 (1) | 1 (2) | 3 (6) | 3 (13) |
| Asthenia | 2 (3) | 2 (5) | 3 (6) | 2 (9) |
| Dizziness | 3 (4) | 1 (2) | 3 (6) | 0 |

All AEs listed are treatment emergent. Adverse Events were coded using MedDRA Version 24.0

AE, adverse event; BL, baseline; MedDRA, Medical Dictionary for Regulatory Activites; NRS, numeric rating scale.

## **Supplemental Figures**

### ***Supplemental Figure 1.*** *RESPONSE study design and schedule of PRO assessments*


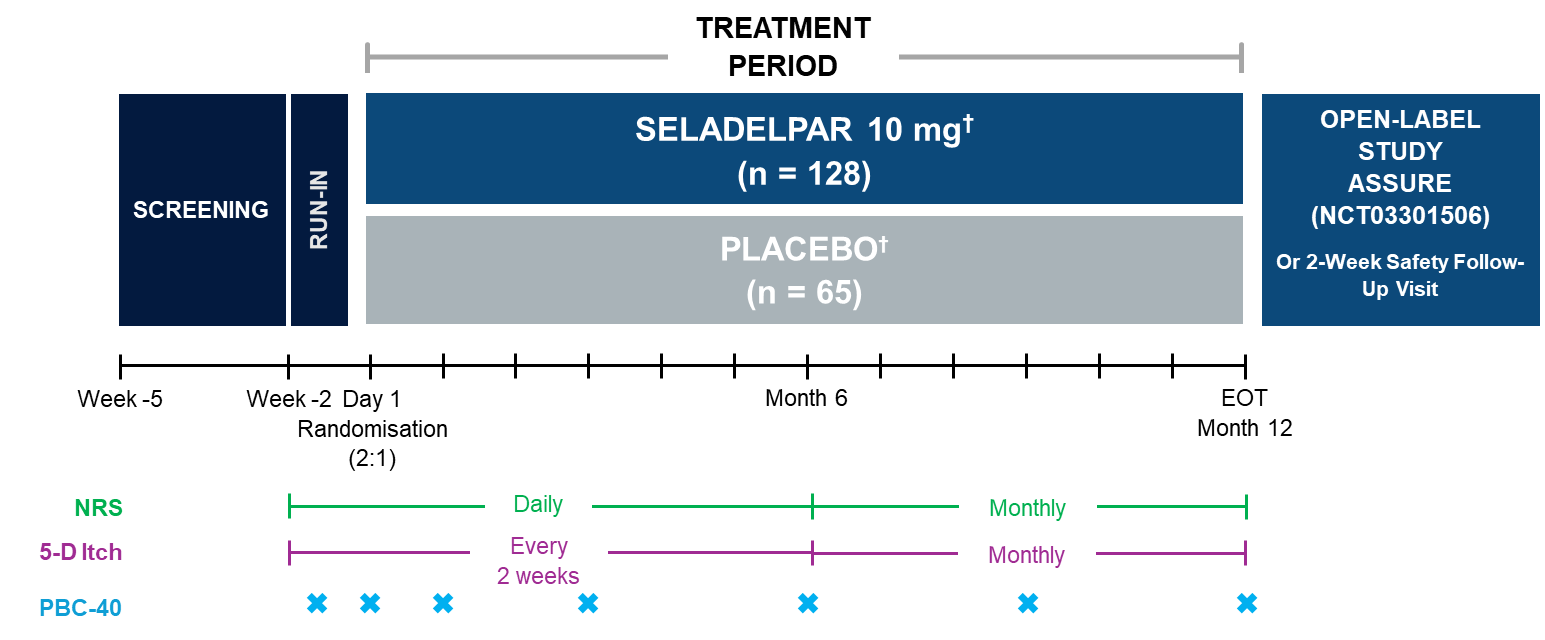


NRS data was collected via e-diary on a daily basis from the run-in visit through the first 6 months of treatment, then monthly for 7 consecutive days each month until EOT. 5-D Itch data were collected every 2 weeks from run-in through 6 months, then monthly through 12 months or until the end of treatment. PBC-40 data were collected at run-in, randomisation, 1 month, 3 months, and then every 3 months through 12 months or until the end of treatment.

**^†^**Study drug given as an add-on to UDCA in patients on UDCA for at least 12 months, or as monotherapy in patients intolerant to UDCA.

EOT, end of treatment; NRS, numeric rating scale; PBC, primary biliary cholangitis; PRO, patient-reported outcome; UDCA, ursodeoxycholic acid.

7

### ***Supplemental Figure 2.*** *5-D Itch changes in all domains and the sleep disturbance item at months 6 and 12 in patients with moderate to severe pruritus based on NRS ≥4 at baseline*


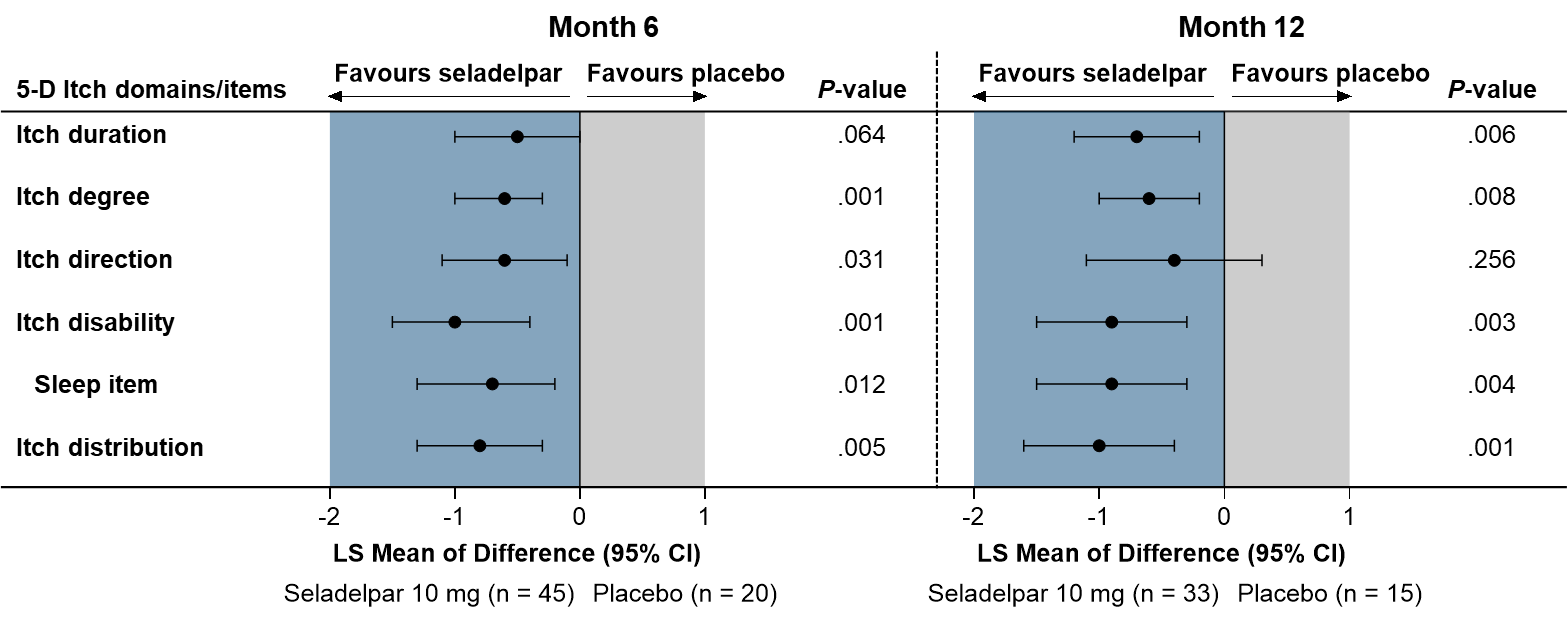


LS mean differences (95% CI) at 6 months were –0.5 (–1.0, 0.0) for duration, –0.6 (–1.0, –0.3) for degree, –0.6 (–1.1, –0.1) for direction, –1.0 (–1.5, –0.4) for disability, –0.7 (–1.3, –0.2) for sleep, and
–0.8 (–1.3, –0.3) for distribution. LS mean differences (95% CI) at 12 months were –0.7 (–1.2,
–0.2) for duration, –0.6 (–1.0, –0.2) for degree, –0.4 (–1.1, 0.3) for direction, –0.9 (–1.5, –0.3) for disability, –0.9 (–1.5, –0.3) for sleep, and –1.0 (–1.6, –0.4) for distribution.

LS, least-square; NRS, numeric rating scale.

### ***Supplemental Figure 3.*** *PBC-40 changes in all domains and the sleep disturbance question at months 6 and 12 in patients with moderate to severe pruritus based on NRS ≥4 at baseline*


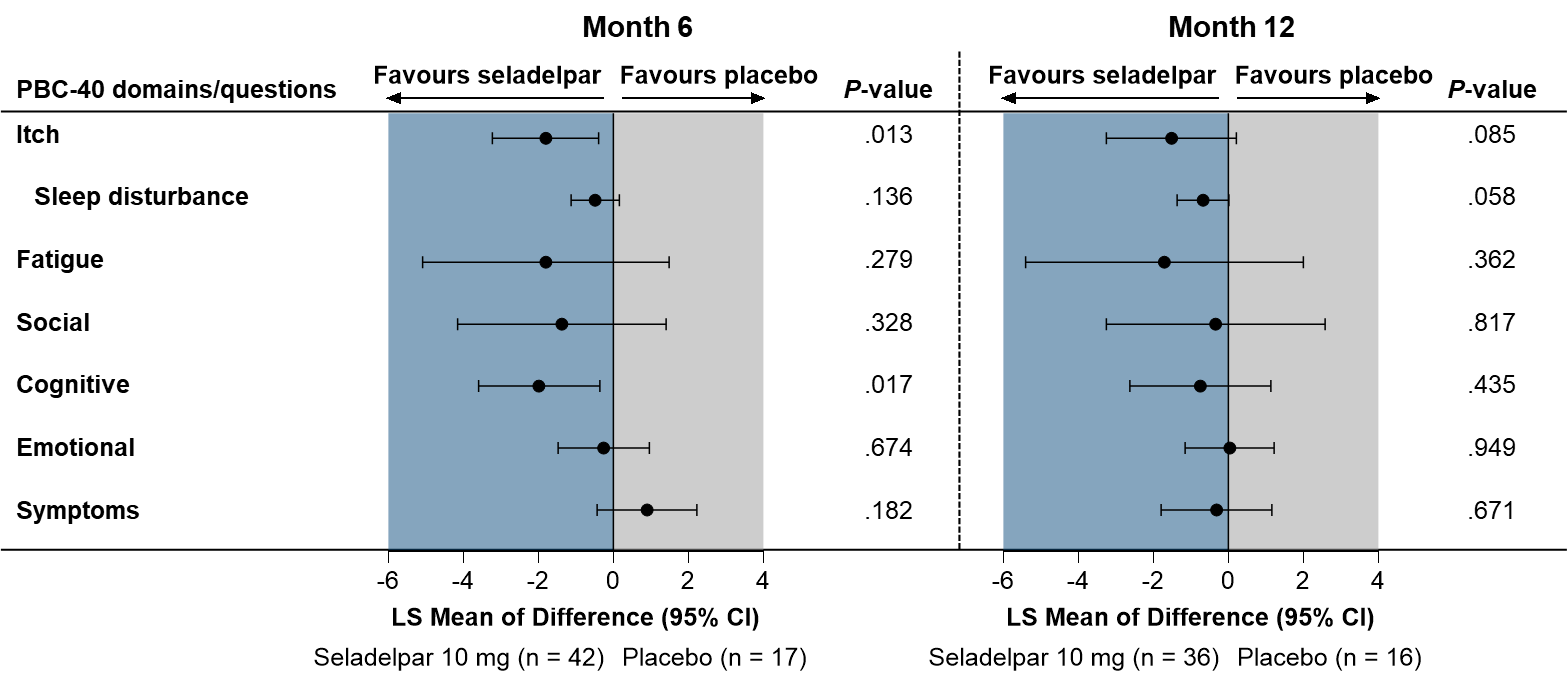


*p* <.05 at months 1, 3, 6, and 9, favouring seladelpar vs placebo for the PBC-40 itch domain in patients with NRS ≥4 at baseline.

LS mean differences (95% CI) at 6 months were –1.8 (–3.2, –0.4) for itch, –0.5 (–1.1, 0.2) for sleep, –1.8 (–5.1, 1.5) for fatigue, –1.4 (–4.2, 1.4) for social, –2.0 (–3.6, –0.4) for cognitive, –0.3 (–1.5, 1.0) for emotional, and 0.9 (–0.4, 2.2) for symptoms. LS mean differences (95% CI) at 12 months were
–1.5 (–3.3, 0.2) for itch, –0.7 (–1.4, 0.0) for sleep, –1.7 (–5.4, 2.0) for fatigue, –0.3 (–3.3, 2.6) for social, –0.7 (–2.6, 1.1) for cognitive, 0.0 (–1.2, 1.2) for emotional, and –0.3 (–1.8, 1.2) for symptoms.

LS, least-square; NRS, numeric rating scale; PBC, primary biliary cholangitis.

### ***Supplemental Figure 4.*** *Spearman correlations of the change in PBC-40 itch and fatigue domain scores at months 6 and 12 in patients with moderate to severe pruritus based on NRS ≥4 at baseline*

(A)


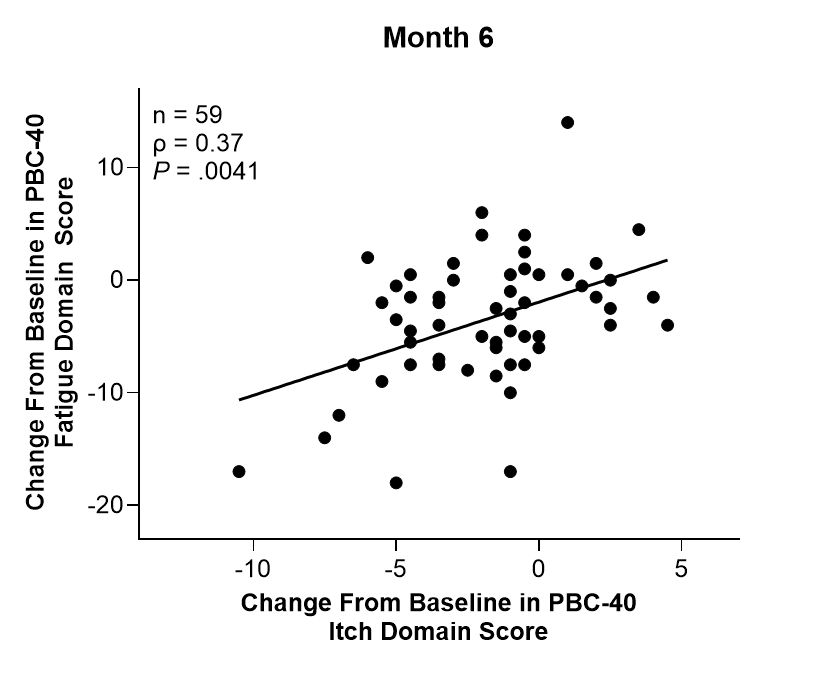


(B)


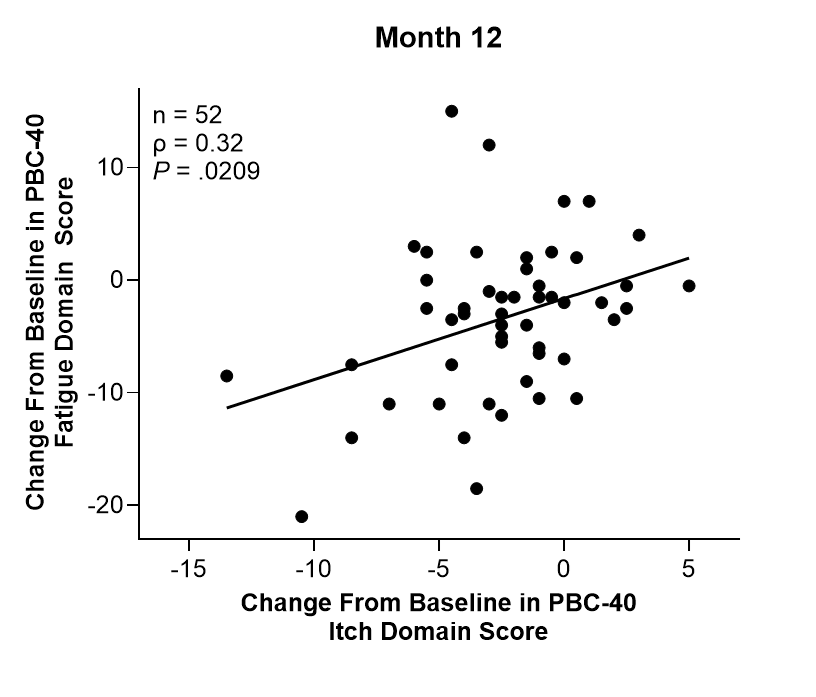


NRS, numeric rating scale; PBC, primary biliary cholangitis.

7

### ***Supplemental Figure 5.*** *5-D Itch changes in all domains and the sleep disturbance item at months 6 and 12 in patients with severe pruritus based on NRS ≥7 at baseline*


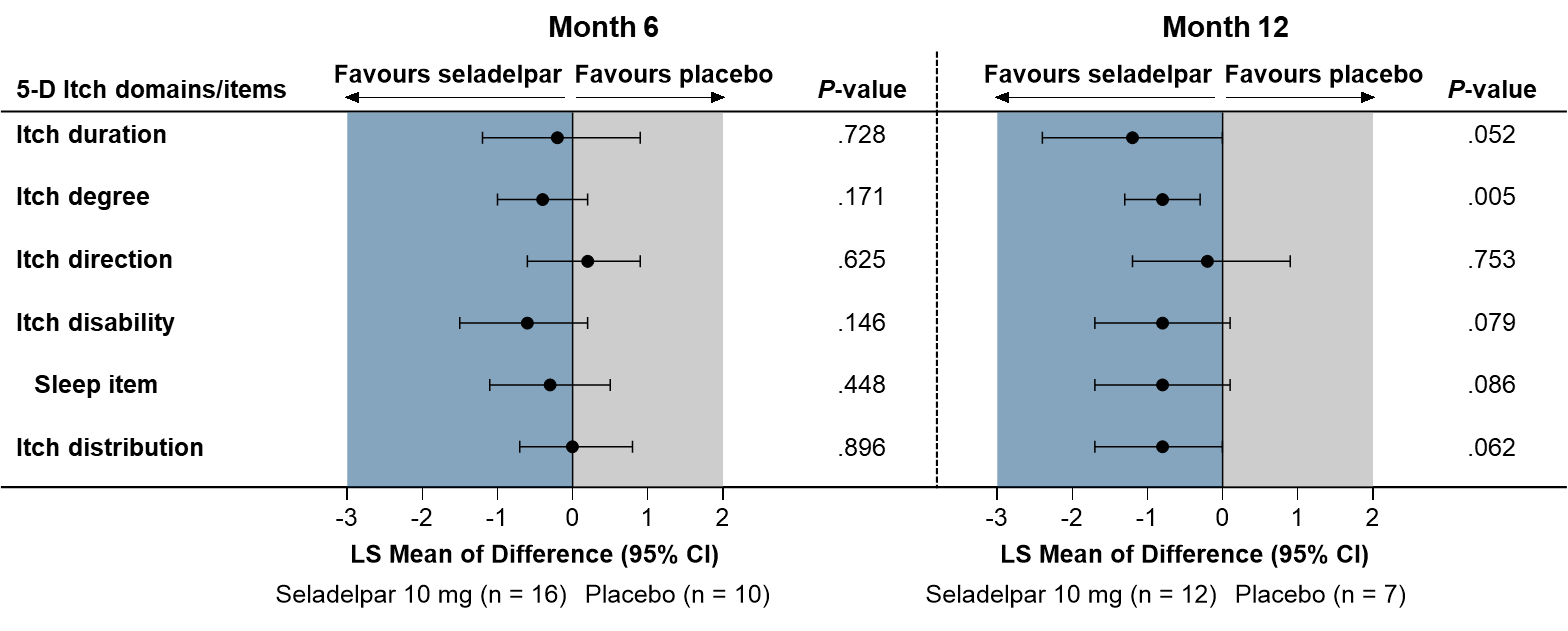


LS mean differences (95% CI) at 6 months were –0.2 (–1.2, 0.9) for duration, –0.4 (–1.0, 0.2) for degree, 0.2 (–0.6, 0.9) for direction, –0.6 (–1.5, 0.2) for disability, –0.3 (–1.1, 0.5) for sleep, and 0.0 (–0.7, 0.8) for distribution. LS mean differences (95% CI) at 12 months were –1.2 (–2.4, 0.0) for duration, –0.8 (–1.3, –0.3) for degree, –0.2 (–1.2, 0.9) for direction, –0.8 (–1.7, 0.1) for disability,
–0.8 (–1.7, 0.1) for sleep, and –0.8 (–1.7, 0.0) for distribution.

LS, least-square; NRS, numeric rating scale.

### ***Supplemental Figure 6****. PBC-40 changes in all domains and the sleep disturbance question at months 6 and 12 in patients with severe pruritus based on NRS ≥7 at baseline*

**
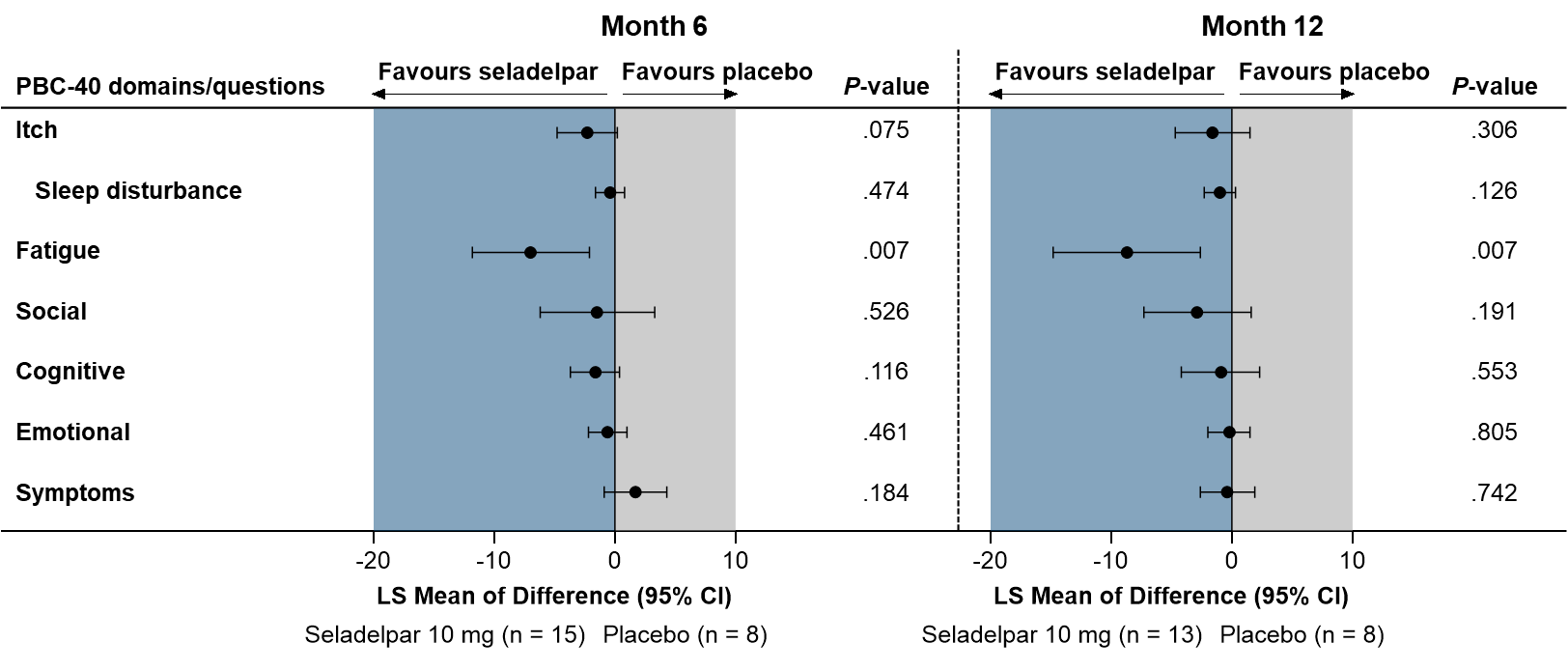
**

LS mean differences (95% CI) at 6 months were –2.3 (–4.8, 0.2) for itch, –0.4 (–1.6, 0.8) for sleep, –7.0 (–11.8, –2.1) for fatigue, –1.5 (–6.2, 3.3) for social, –1.6 (–3.7, 0.4) for cognitive, –0.6 (–2.2, 1.0) for emotional, and 1.7 (–0.9, 4.3) for symptoms. LS mean differences (95% CI) at 12 months were –1.6 (–4.7, 1.5) for itch, –1.0 (–2.3, 0.3) for sleep, –8.7 (–14.8, –2.6) for fatigue, –2.9 (–7.3, 1.6) for social, –0.9 (–4.2, 2.3) for cognitive, –0.2 (–2.0, 1.5) for emotional, and –0.4 (–2.6, 1.9) for symptoms.

LS, least-square; NRS, numeric rating scale; PBC, primary biliary cholangitis.

### ***Supplemental Figure 7****. PBC-40 changes in all domains and the sleep disturbance question at months 6 and 12 in patients with clinically significant pruritus based on PBC-40 itch ≥7 at baseline*

**
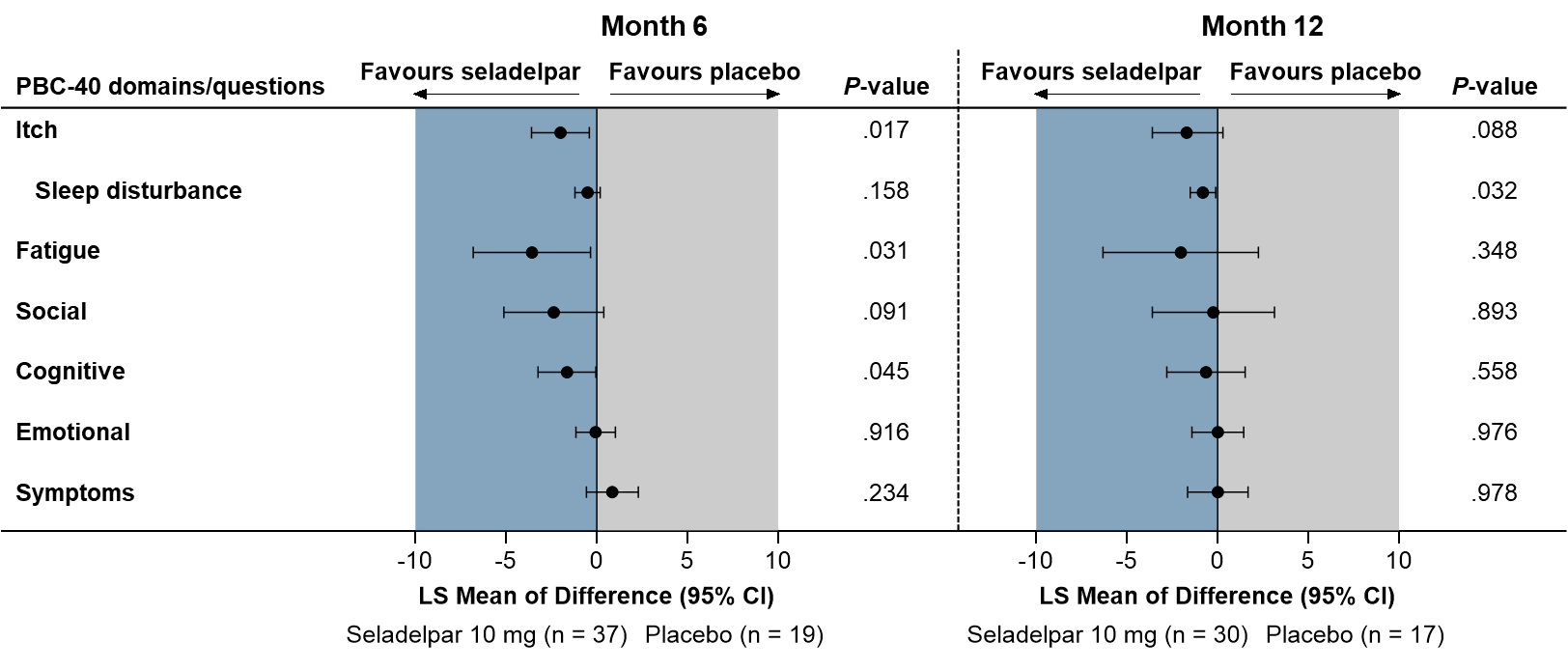
**

LS mean differences (95% CI) at 6 months were –2.0 (–3.6, –0.4) for itch, –0.5 (–1.2, 0.2) for sleep, –3.6 (–6.8, –0.3) for fatigue, –2.4 (–5.1, 0.4) for social, –1.6 (–3.2, 0.0) for cognitive, –0.1 (–1.1, 1.0) for emotional, and 0.9 (–0.6, 2.3) for symptoms. LS mean differences (95% CI) at 12 months were –1.7 (–3.6, 0.3) for itch, –0.8 (–1.5, –0.1) for sleep, –2.0 (–6.3, 2.3) for fatigue, –0.2
(–3.6, 3.1) for social, –0.6 (–2.8, 1.5) for cognitive, 0.0 (–1.4, 1.5) for emotional, and 0.0 (–1.6, 1.7) for symptoms.

LS, least-square; PBC, primary biliary cholangitis.

### ***Supplemental Figure 8****. Mean pruritus NRS over time in patients with moderate to severe pruritus based on NRS ≥4 at BL (colour-blind accessible)*


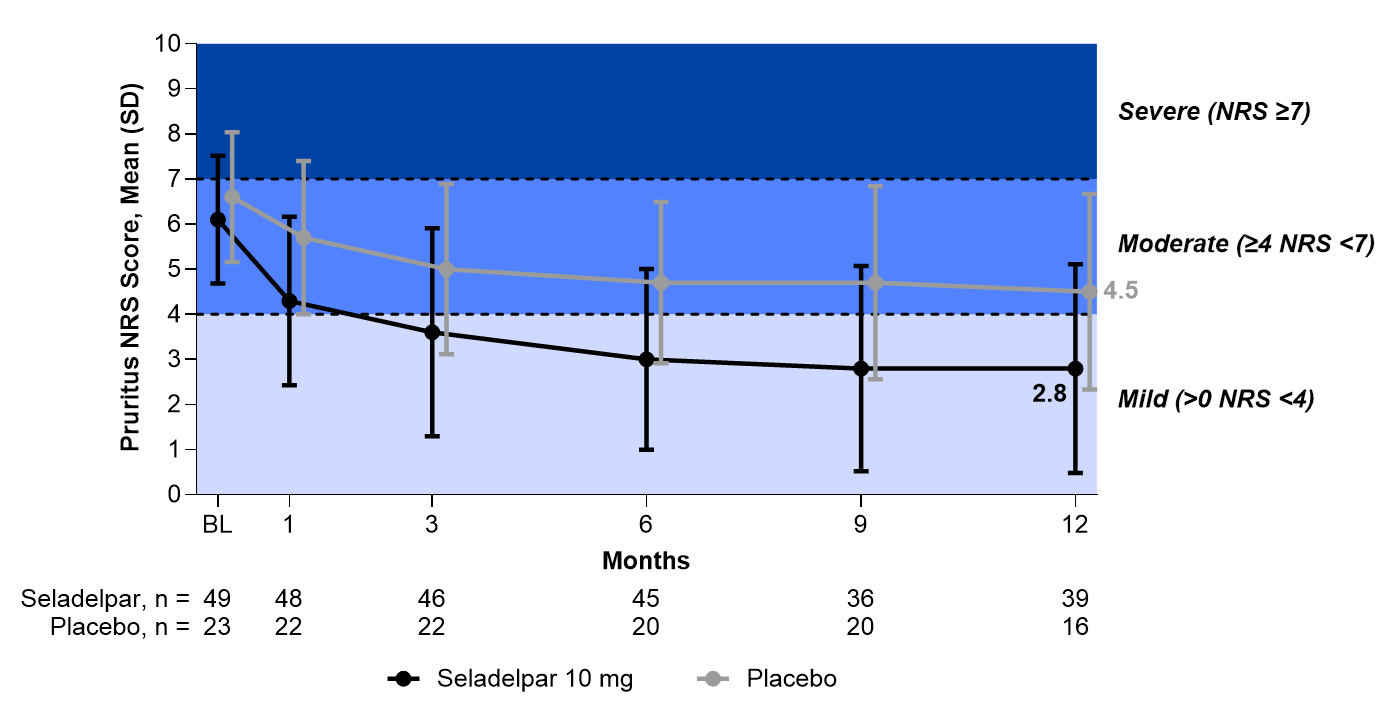


n = the number of patients who had both a BL value and a value at that time point.

BL, baseline; NRS, numeric rating scale.

### ***Supplemental Figure 9.*** *Daily* *duration of itch (a) and body regions with pruritus (b) over time based on 5-D Itch in patients with moderate to severe pruritus based on NRS ≥4 at baseline (colour-blind accessible)*

(A)


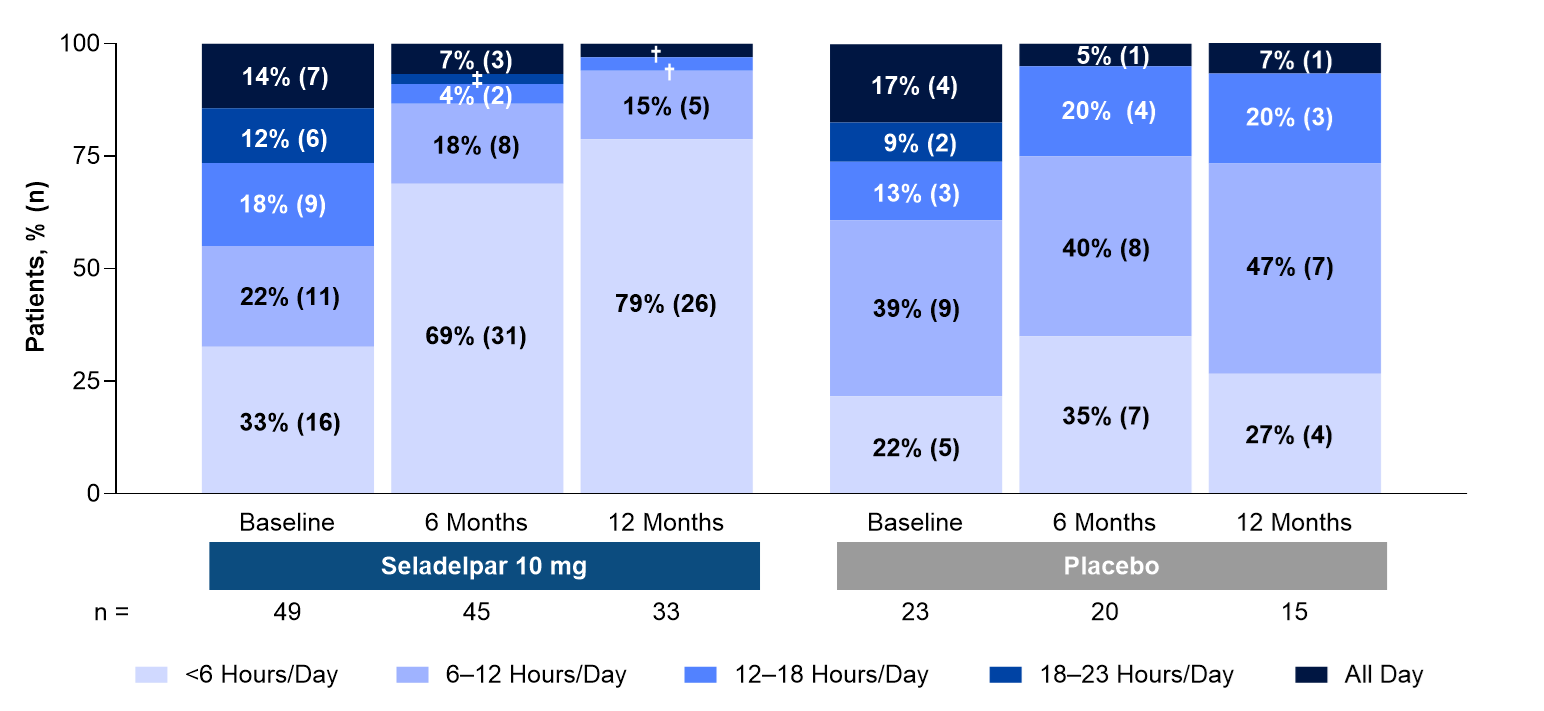


(B)


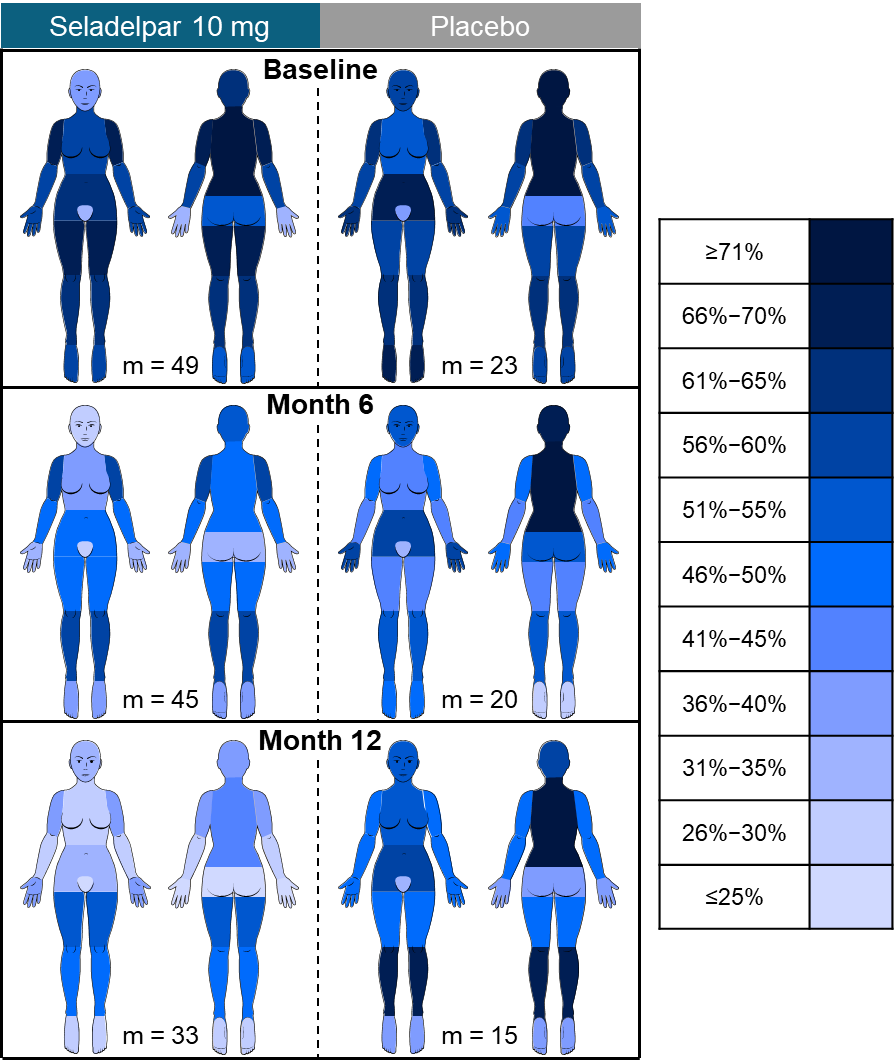


^‡^In panel A, the symbol represents 2% (1).

^†^In panel A, the symbol represents 3% (1).

In panel A, n = the number of patients who had both a baseline value and a value at that time point.

In panel B, percentages represent the number of patients who reported itching in that body region at that analysis visit divided by the number of patients who had a value in that body region at that analysis visit (m).

NRS, numeric rating scale.

### ***Supplemental Figure 10.*** *Sankey plot of baseline and month 12 NRS categories for seladelpar and placebo in overall study population (colour-blind accessible)*


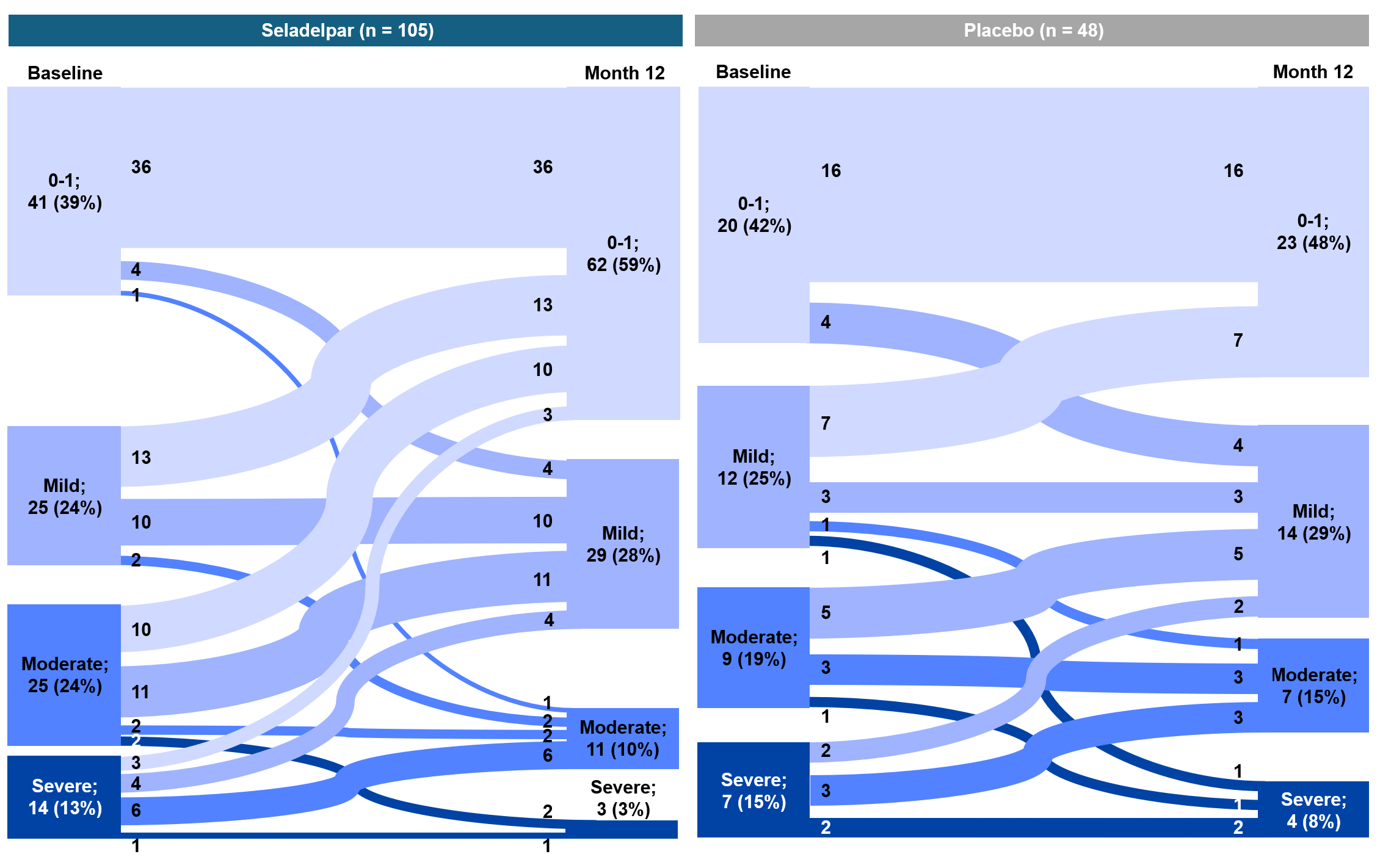


Categories were defined as: no or near no itch (NRS = 0–1), mild (NRS >1 to <4), moderate (NRS 4 to <7), or severe itch (NRS ≥7). Includes patients with data available at both baseline and 12 months.

NRS, numeric rating scale.

# References

1. Kremer AE, Mayo MJ, Hirschfield G, et al. Seladelpar improved measures of pruritus, sleep, and fatigue and decreased serum bile acids in patients with primary biliary cholangitis. *Liver Int*. Jan 2022;42(1):112-123. doi:10.1111/liv.15039

2. Elman S, Hynan LS, Gabriel V, Mayo MJ. The 5-D itch scale: a new measure of pruritus. *Br J Dermatol*. Mar 2010;162(3):587-93. doi:10.1111/j.1365-2133.2009.09586.x

3. Jacoby A, Rannard A, Buck D, et al. Development, validation, and evaluation of the PBC-40, a disease specific health related quality of life measure for primary biliary cirrhosis. *Gut*. Nov 2005;54(11):1622-9. doi:10.1136/gut.2005.065862

4. Al-Harthy N, Kumagi T, Coltescu C, Hirschfield GM. The specificity of fatigue in primary biliary cirrhosis: evaluation of a large clinic practice. *Hepatology*. Aug 2010;52(2):562-70. doi:10.1002/hep.23683
